# Chrombus-XMBD: a graph convolution model predicting 3D-genome from chromatin features

**DOI:** 10.1093/bib/bbaf183

**Published:** 2025-05-02

**Authors:** Yuanyuan Zeng, Zhiyu You, Jiayang Guo, Jialin Zhao, Ying Zhou, Jialiang Huang, Xiaowen Lyu, Longbiao Chen, Qiyuan Li

**Affiliations:** Department of Hematology, The First Affiliated Hospital of Xiamen University and Institute of Hematology, School of Medicine, Xiamen University, Xiamen, Fujian 361102, China; National Institute for Data Science in Health and Medicine, School of Medicine, Xiamen University, Xiamen, Fujian 361102, China; National Institute for Data Science in Health and Medicine, School of Medicine, Xiamen University, Xiamen, Fujian 361102, China; National Institute for Data Science in Health and Medicine, School of Medicine, Xiamen University, Xiamen, Fujian 361102, China; National Institute for Data Science in Health and Medicine, School of Medicine, Xiamen University, Xiamen, Fujian 361102, China; National Institute for Data Science in Health and Medicine, School of Medicine, Xiamen University, Xiamen, Fujian 361102, China; State Key Laboratory of Cellular Stress Biology, School of Life Sciences, Faculty of Medicine and Life Sciences, Xiamen University, Xiamen, Fujian 361102, China; State Key Laboratory of Cellular Stress Biology, Fujian Provincial Key Laboratory of Reproductive Health Research, School of Medicine, Xiamen University, Xiamen, Fujian 361102, China; Fujian Key Laboratory of Sensing and Computing for Smart Cities (SCSC), School of Informatics, Xiamen University, Xiamen, Fujian 361102, China; Department of Hematology, The First Affiliated Hospital of Xiamen University and Institute of Hematology, School of Medicine, Xiamen University, Xiamen, Fujian 361102, China; National Institute for Data Science in Health and Medicine, School of Medicine, Xiamen University, Xiamen, Fujian 361102, China

**Keywords:** 3D-genome, graph autoencoder, *de novo* prediction, epigenomic features, long-range interaction, model generalizability

## Abstract

The 3D conformation of the chromatin is crucial for transcriptional regulation. However, current experimental techniques for detecting the 3D structure of the genome are costly and limited to the biological conditions. Here, we described “Chrombus^XMBD^,” a graph convolution model capable of predicting chromatin interactions *ab initio* based on available chromatin features. Using dynamic edge convolution with multihead attention mechanism, Chrombus encodes the 2D-chromatin features into a learnable embedding space, thereby generating a genome-wide 3D-contactmap. In validation, Chrombus effectively recapitulated the topological associated domains, expression quantitative trait loci, and promoter/enhancer interactions. Especially, Chrombus outperforms existing algorithms in predicting chromatin interactions over 1–2 Mb, increasing prediction correlation by 11.8%–48.7%, and predicts long-range interactions over 2 Mb (Pearson’s coefficient 0.243–0.582). Chrombus also exhibits strong generalizability across human and mouse-derived cell lines. Additionally, the parameters of Chrombus inform the biological mechanisms underlying cistrome. Our model provides a new, generalizable analytical tool for understanding the complex dynamics of chromatin interactions and the landscape of *cis*-regulation of gene expression.

## Introduction

The 3D conformation of chromatin, or 3D-genome, plays a crucial role in the organization and regulation of transcription [1]. Formation of DNA loops mediated by specific epigenetic modifications and *trans*-acting proteins enables the sophisticated *cis*-regulation of gene expression in eukaryote cells, such as enhancer–promoter and enhancer–enhancer interactions [2, 3]. Techniques such as 3C [4], 4C [5], 5C [6], Hi-C [7], ChIA-PET [8], and HiChIP [9] have been developed to capture the landscape of DNA interactions either at a specific locus or a genome-wide level. These interactions can be used to interpret the biological processes underlying known trait-associated loci (TALs), such as risk loci from genome-wide association studies (GWAS) and eQTL, which are widely implicated in development, differentiation, and diseases [10–13].

The most common structural elements of 3D-genome are DNA loops [14–17]. At greater scales, chromosomes are organized into topologically associating domains (TADs), compartments, and chromosome territories [18, 19]. According to the loop extrusion hypothesis, the formation of DNA loops requires two CCCTC-Binding Factor (CTCF) binding sites in opposite orientations. Cohesin actively slides along the chromatin fiber and extrude it into a circular conformation [20–23]. Besides, certain histone modifications are also involved in chromatin interactions, which enable transcription regulation via enhancer-promoter (E-P), enhancer-enhancer (E-E), and promoter–promoter (P-P) loops [24–26].

To date, our understanding of the 3D-genome is primarily based on Hi-C experiments conducted in cell lines or tissues. Many computational methods have been developed to resolve the 3D-genome from Hi-C data thus infer the *cis*-regulatory programs of eukaryote cells. Nevertheless, most of the studies are still based on limited sample size, and the algorithms often lack consistency, which hinders the understanding of 3D-genome organization under more diverse biological contexts [27]. Recent studies use machine learning methods to predict 3D-genome from chromatin features, which provide an alternative approach to solve the complex chromatin conformation. Akita [28] and DeepC [29] predict locus-specific genome folding based on DNA sequence. HiC-Reg [30] and Epiphany [31] predict Hi-C-based contact counts using chromatin features. C. Origami [32] predicts chromatin interaction intensities using both sequence and chromatin features. C. Origami is capable of predicting Chromatin interactions up to 2 Mb compared to 1 Mb by the other methods. Most of the existing algorithms use the convolutional neural network (CNN) except for HiC-Reg, which is based on Random Forest. Epiphany adopts more complex architectures such as LSTM and GAN, and C. Origami uses Transformer. Nevertheless, CNN-based algorithms require fixed genome bin sizes, which makes them difficult to adapt to genomic data at varying scales [28, 29]. GANs often face training instability, leading to challenges in model generalizability [31]. LSTMs and Transformer require substantial computational resources, particularly when processing genomic data [31, 32]. These limitations constrain the predicted interactions by the current methods to a maximal 2 Mb range. Then, real chromatin segments are unevenly distributed, and the interactions between two DNA segments can be influenced by chromatin features of their distant neighbors [14, 33]. Finally, the lack of interpretability further hinders the application of deep-learning methods in functional research.

Many complex biological interactions are represented as graphs [34–36]. The graph neural network (GNN) provides a framework for knowledge inference on graphs, which is successfully applied to drug discovery, protein conformation, and gene expression analysis [37, 38]. The advantages of GNN in modeling biological systems rely on its capability to process non-Euclidean, complex interactions among biological entities.

Here we described a new, graph-based model, “Chrombus,” which is capable of *ab initio* prediction of the 3D conformation of chromatin based on specific epigenetic features. Our algorithm aggregates signals from a set of neighboring CTCF-based segments and predicts the interaction potential, thereby revealing context-specific formation of TADs. Of note, our graph-based algorithm showed highly generalizable predictive power and was capable of predicting long-range DNA interactions, which outperformed the state-of-the-art methods [28–31].

We rigorously validated our method against known evidence of 3D-genome, including eQTL associations and enhancer–gene architectures. We demonstrated that “Chrombus” can recover known *cis*-regulatory events and predict regulatory programs highly relevant to biology. Our method provides an efficient, robust way to reconstruct the landscape of 3D-genome at all levels.

## Material and methods

### Graph representation of 3D-genome based on CTCF-segments

**Figure 1:**
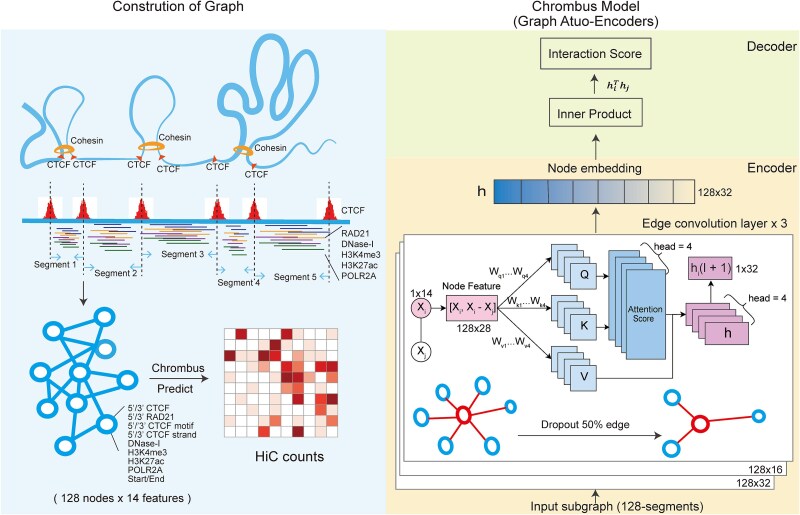
Graph representation of 3D-genome and “Chrombus” auto-encoder architecture. Each subgraph consists 128 vertices, and each vertex represents a chromatin segment derived from CTCF binding peaks. The node (vertex) attributes consist 14-dimensional chromatin features. The goal of the learning process is to predict the real-valued interaction strength among vertices, of which the labeling is based on preprocessed Hi-C data (left panel). Chrombus is adopted from GAE architecture. The encoder consists of three edge convolution layers with embedded attention mechanism and outputs embedding of dimensions 32, 16, and 32. The decoder is implemented as a plain inner product (right panel).

We segmented the chromatin based on CTCF-binding peaks. Each fragment corresponds to a vertex, represented by 14-dimension feature vector ${\boldsymbol{x}}_{\boldsymbol{i}}$, denoted as ${\boldsymbol{V}}_{\boldsymbol{i}}\mathbf{\in}\boldsymbol{V}$ (Supplementary Table 2, Supplementary Figs 26–31). Then, the edge, ${\boldsymbol{E}}_{\boldsymbol{ij}}$, between the vertices *i* and *j* were defined by the averaged interaction score between the two fragments derived directly from processed Hi-C data. Thus, the 3D structure of the chromatin is represented by a graph $\boldsymbol{G}\left(\boldsymbol{V},\boldsymbol{E}\right)$ (Supplementary Methods, Fig. 1).

It ought to be mentioned that during inference, Chrombus takes batched, randomly sampled consecutive CTCF segments without edge information, and the edge information is only used for loss function.

### The Chrombus model

“Chrombus” is a graph convolution model that reconstructs chromatin interaction (Fig. 1). Chrombus adopts a typical Graph Autoencoder (GAE) [39] model. The encoder maps 14-dimensional input features of a graph to 32-dimensional representation $\boldsymbol{z}=\left({z}_i,\dots, {z}_N\right)$ through 3-depth edge-convolution layers [eq. (1)], and the decoder generates *N*-by-*N* matrix of interaction strength as $\boldsymbol{A}={\boldsymbol{z}}^{\prime}\boldsymbol{z}$ (Supplementary Methods).


$$ {\mathrm{h}}_i^{(0)}={\mathrm{W}}^0\cdot \left[{\mathrm{x}}_j^{(0)}-{\mathrm{x}}_i^{(0)}\Big\Vert{\mathrm{x}}_i^{(0)}\right]+{\mathrm{b}}^0 $$

(1) \begin{equation*} {\mathrm{h}}_i^{l+1}=\mathrm{\sigma} \left(\sum_{j\in N(i)}{a}_{ij}^l\left[{\mathrm{W}}^l\cdot \left[{\mathrm{h}}_j^l-{\mathrm{h}}_i^l|\Big|{\mathrm{h}}_i^l\right]+{\mathrm{b}}^l\right]\right) \end{equation*}

where ${\mathrm{W}}^l$ and ${\mathrm{b}}^l$ denotes the trainable weights and bias of the $l$-th layer, $\mathrm{\sigma}$ represents the sigmoid activation function, $N(i)$ represent the neighbors of node $i$, and $i$ and *j* represent the indices of target and source nodes, respectively. ${a}_{ij}^l$ is a partition matrix that gathers and reallocates the weight of hidden representations from $N(i)$ in the $l$-th layer.

To ensure the target node receives relevant information from its random neighbors during edge convolution (${a}_{ij}^l$), we employed a multihead (*n* = 8) self-attention as described in the transformer model [40] (Supplementary Methods). In addition, we designed a signed edge weight to address the biological fact that interaction between segments is negatively correlated with the linear distance (Supplementary Methods). We defined a *cis*-interaction range of nine consecutive segments so that the sign of $\omega$ flips according to ${D}_{ij}$, the linear distance between two segments measured by the number of segments in between [eq. (2)].


(2) \begin{equation*} {\omega}_{ij}=\log \left(\frac{D_{ij}\mid{Index}_i-{Index}_j\mid }{9}\right) \end{equation*}

To train “Chrombus,” the encoder takes batched subgraphs as input. Each subgraph contains 128 consecutive CTCF-segments that were randomly cropped from a chromatin. During training and testing, the vertices were connected by random edges based on the following rules: (i) no interactions beyond the maximal span of 64 consecutive CTCF-segments, and (ii) within the maximal span, any pair of CTCF-segments are connected by 50% chance, which correspond to an Erdős–Rényi random graph of G (128, 0.5). The objective of the training is to minimize the MSE between predicted and real interactions (edge attributes).

We employed a “leave-one-out” model on GM12878. The dataset was randomly put-back cropped into 128-segments from one chromosome as testing chromosome, and the other 21 chromosomes were used for training (Supplementary Methods).

### Model evaluation

To validate “Chrombus,” we compared predicted chromatin interactions between two segments within a defined TAD (“Within TAD”), and those between two segments located in different adjacent TADs (“Between TADs”). We used receiver operating characteristic (ROC) curves and the area under the curve (AUC) to assess the distinguishability of the predicted interaction scores between the two groups. In addition, we evaluated the correlation between TAD separation scores derived based on Chrombus prediction and the true Hi-C data.

Then, we compared the predicted interactions with independent datasets of eQTL interactions and enhancer–gene interactions and evaluated the enrichment of known genomic interactions in the prediction by Chrombus (Supplementary Methods).

Finally, we compared the performance of Chrombus with baseline model and two other existing methods, Epiphany and C. Origami.

### Feature importance and node representation

The input features’ contributions in predicting chromatin interaction are computed via the GNNexplainer algorithm. The node mask value is learned using PyTorch genometric (v2.3.1) with 200 epochs. The correlation between node representation and input feature is determined using the Pearson correlation coefficient.

## Results

### Chrombus: accurate prediction of chromatin interactions using epigenetic features through a graph neural network

We developed “Chrombus,” a graph-based model to predict chromatin interactions *ab initio* based on epigenetic features (Fig. 1, Methods). “Chrombus” uses three dynamic edge-convolution layers with multihead attention and is trained with multilevel epigenetic features (input) and Hi-C data (label) from the lymphoblastoid cell line (GM12878). Instead of using evenly distributed bins of the chromatin, we used 39 975 CTCF-peaks to define segments of DNA as units of interactions. A typical dataset contains 39 998–59 443 such segments and 6–21 million positive interactions (Supplementary Figs 1–3).

The training data consist of batched subgraphs of 128 adjacent CTCF-segments that were randomly cropped from the contact map (Supplementary Methods). In each round of training, 1 of 22 autosomes is left out as test data with the rest as training data. To evaluate the prediction performance, the training–test process is repeated for 22 rounds, with each autosome being tested independently based on the model trained on the others, resulting in 22 models.

**Figure 2:**
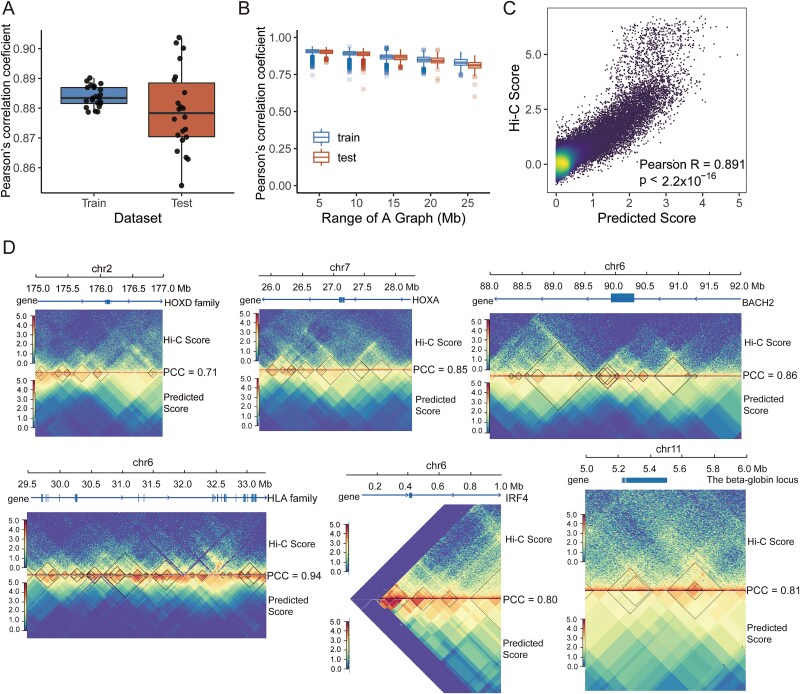
Chrombus recovered known chromatin interactions and TADs. The model parameters were trained and tested independently using chromatin features of GM12878. The goodness-of-fit between the true Hi-C scores and the predicted scores was evaluated using Pearson’s correlation coefficient (PCC). (A) Comparison of the Pearson’s correlation coefficients between the true Hi-C scores and the predicted values of Chrombus for each autosome in the training and testing. (B) Pearson’s correlation coefficients between the true Hi-C scores and the predicted scores of the subgraphs of different ranges in the training and testing datasets. The subgraphs were grouped by the range (Mb). (C) Scatter plot showing the correlation between the predicted values and true Hi-C scores in 100 000 randomly selected test loci. (D) Chrombus predicted chromatin interactions at six known TAD loci. The arrows indicating the directions of the genes. The PCCs between the true Hi-C scores and predicted scores range from 0.71 to 0.94.

Each model reached convergence after ~400 epochs. Both the training losses and the testing losses followed the same trend during the training process (Supplementary Fig. 4A). We evaluated the performance of “Chrombus” using Pearson’s correlation coefficients between the predicted scores and the actual Hi-C scores. For the training data, all 22 models converged with correlation coefficients ranging from 0.880 to 0.893 and the mean square error (MSE) ranging from 0.140 to 0.162 (Fig. 2A, Supplementary Fig. 4A). For the testing data, the correlation coefficients ranging from 0.849 to 0.900 and the MSE ranging from 0.126 to 0.286 (Fig. 2A, Supplementary Fig. 4A). We also noticed that the training correlation and testing correlation decreased slightly with the range of each subgraph (Fig. 2B). Particularly, our data showed that “Chrombus” performed better with CTCF-based segmentations than even-sized bins, highlighting the advantage of biologically informed chromatin segmentation over conventional partitioning (Supplementary Fig. 5).

We further estimated the overall correlation between predicted scores and Hi-C scores in 100 000 (2.169%) randomly sampled pairs of segments from all 22 autosomes and yielding a correlation coefficient of 0.891 (0.889–0.892, 95% CI) (Fig. 2C).

To cope with the distance and size effects of 3D-genome, we devised a contrast edge weight (*n* = 9) based on the average number of segment counts within TADs. When two segments are separated by more than nine other segments, self-attention assigns positive weights to stronger interactions. Conversely, when two segments are located within nine segments, self-attention assigns negative weights to stronger interactions. This approach enhances the model’s sensitivity to long-distance interactions, which hold greater biological significance, and dissociation events that occur between segments at shorter distances, commonly found at the boundaries of TADs or compartments. The parameter setting (*n* = 9) was optimized from 3 to 21 (Supplementary Table 1, Supplementary Fig. 4B and C). Additionally, we optimized the number of heads and tested the effectiveness of multihead attention. The results demonstrated that multihead (*N* = 8) improved the model’s predictive performance (Supplementary Fig. 6). From the testing results, we selectively examined a set of known TADs in the GM12878 cell line, including IRF4 [41], BACH [42], HOXD family [43], HLA family [44], HOXA family [45], and the beta-globin locus [46] (Fig. 2D). These TADs spanned various regions of genome, and the prediction of Chrombus highly correlated to the corresponding Hi-C scores, with Pearson’s correlation coefficients ranging from 0.71 to 0.94 (Supplementary Fig. 7). The results suggest that Chrombus is capable of recovering known TADs with high consistency.

In addition, we compared Chrombus to three GNN architectures for predicting binary statuses of chromatin interaction, including Dynamic Edge Convolutional (DynamicEdgeConv) [47], graph attention network (GAT) [48], and graph convolutional network (GCN) [49]. The results demonstrated that Chrombus outperformed the baseline models in F1-score and balanced accuracy (Supplementary Fig. 3C–E).

### Chrombus prediction unveils chromatin features influencing the formation of 3D-genome

To understand the impact of chromatin features on Chrombus’ prediction of chromatin interactions, we calculated the importance of all input features using GNNexplainer [50]. Overall, chromatin segmentation (3′CTCF, 5′CTCF), relative positioning of the segments (Start and End), chromosomal accessibility (DNase-I), transcription activation mark (POLR2A), promoter mark (H3K4me3), and enhancer mark (H3K27ac) are more important than the other input features for the prediction performance of Chrombus, which is consistent with known biology (Supplementary Figs 8 and 9). Moreover, we also noticed that some input features showed different importance among interactions of different ranges. For example, DNase-I, H3K27ac, and CTCF motif binding (5′−/3′-CTCF motif) are more important for short-range interactions (0–1 Mb), whereas H3K4me3 is more important for long-range interactions (>2 M) with square root of Vanilla Coverage (SQRTVC) normalization (Supplementary Fig. 9B). We also noticed that the importance of features was influenced by the normalization methods, such as H3K4me3, POLR2A, and DNase-I (Supplementary Fig. 9A and B).

**Figure 3:**
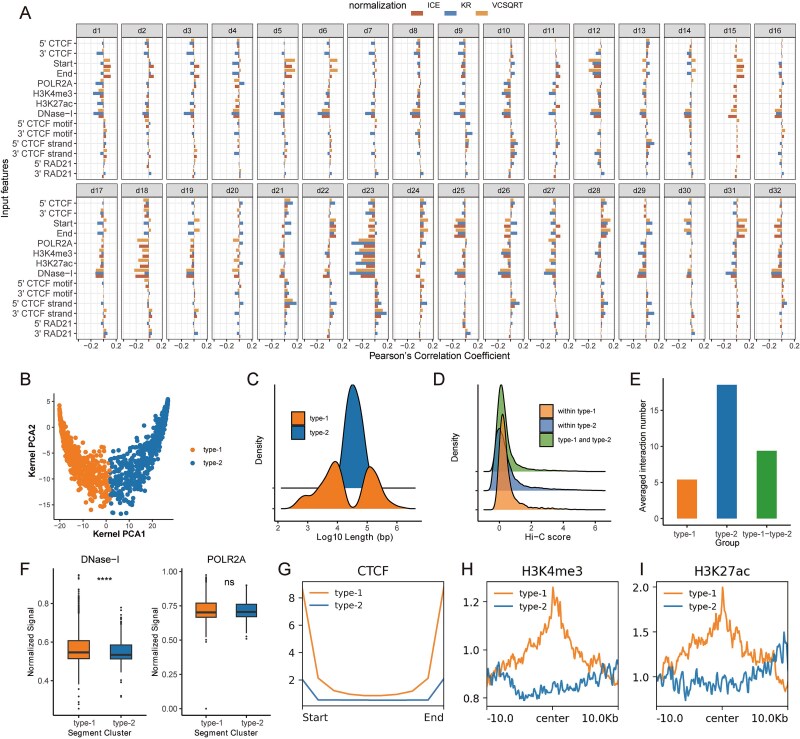
The latent space of Chrombus reflect unique epigenomic features of 3D-genome. Chrombus is trained on GM12878 dataset. Node embeddings were derived from the third (last) layer of edge convolution, which contained 32 dimensions (d1, d2, …, d32). (A) The node embedding correlates with input features. The heights of the bar plots indicate the Pearson’s correlation coefficients between the node embedding and each input node feature, with the color denoting the three normalization methods used. (B) Clustering of node embedding based on the first two kernel PCs reveal two types of segments. (C) Distribution of segment lengths for the type 1 and 2 segments. D: Interaction strengths within type-1, type-2, and between type-1 and type-2 are differently distributed. (E) Numbers of interactions within type-1, type-2, and between type-1 and type-2 are different. (F) DNase-I and POLR2A activities between the two types of segments show trivial differences. (G–I) CTCF, H3K4me3, and H3K27ac landscape at the 10 kb regions flanking the center show distinct patterns for the two types of segments.

We further calculated the Pearson correlation between the node embeddings and the input node features and found that most dimensions have strong correlations with DNase-I and relative positioning of segments (Start and End) (Fig. 3A). Other dimensions tend to represent more specific chromatin features. For example, dimension 23 (d23) showed strong correlation with transcription activation marks (POLR2A, H3K4me3, H3K27ac), whereas CTCF motif binding strand (CTCF-strand) is represented in d10, d21, and d23. Different dimensions of node embedding also reflect the difference among normalization methods. For instance, d15, d18, and d32 of the Knight-Ruiz (KR) normalization show weak correlation with input features (Fig. 3A).

We further investigated the latent space of Chrombus to better understand its predictive power. We extracted the top two principal components of the trained high-dimensional embeddings of CTCF-segments and stratified the CTCF-segments into two distinct categories [51], namely, type-1 and type-2 (Fig. 3B, Methods). Of note, these clusters of segments exhibited marked differences in segment length, interactive activities, and epigenetic features (Fig. 3C–I). In GM12878, type-1 segments show slightly stronger interactions than type-2, and higher DNase-I activities. Then, type-1 also enriches for histone marks of promoter (H3K4me3) and enhancer (H3K27ac), and strong CTCF-peaks at both ends. On the other hand, type-2 segments show less strong epigenetic characteristics but are more interactive as shown by the average number of interactions.

Overall, our data showed that Chrombus extracts relevant information from the input features, hence its interpretability, which can be used to complement the limitations of similar methods in predicting chromatin interactions.

### Chrombus recovers DNA–DNA interaction landscape as represented by topologically associating domains

TADs are known as the elementary 3D structure of chromatin with a crucial role in transcriptional regulation [52]. TADs are isolated from each other at the boundary ends. More active DNA–DNA interactions occur within the TAD than between, which results in topology-associated transcription regulation of genes. Leveraging this distinctive feature, we further verified Chrombus predictions within and between TADs.

**Figure 4:**
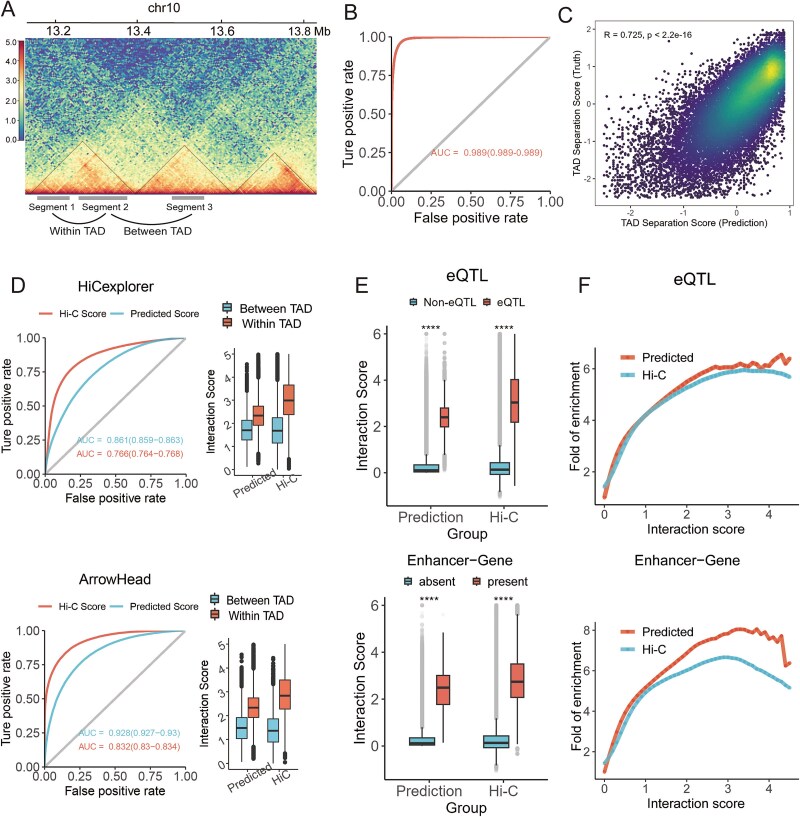
Validation of Chrombus predictions by published datasets of TADs, eQTLs, and enhancer–gene interactions. (A) Illustration of interactions within- and between-TAD (left). (B) ROC curve of binary chromatin interaction statuses based on Chrombus prediction (methods) and true Hi-C scores. The threshold for binarizing Hi-C scores is 1.4. (C) TAD separation scores based on the Chrombus prediction correlated with those based on the true Hi-C scores. (D) ROC curves for classifying chromatin interactions within- and between-TAD based on Chrombus predicted scores and the true Hi-C scores (left), along with interaction strengths in both groups (right). The reference TADs in GM12878 were inferred from the same Hi-C dataset using HiCexplorer (top) and Arrowhead (bottom). (E) Chrombus predictions and the true Hi-C scores are consistently significantly higher in segments encompassing known eQTL–eGENE pairs (top) and enhancer–gene interactions (bottom). (F) Enrichment of eQTL-eGENE (top) and enhancer-genes (bottom) at various levels of interaction scores based on either Chrombus prediction or processed Hi-C data.

We opted for two methods, “HiCexplorer” and “Arrowhead,” to define TADs from Hi-C data (Supplementary Methods). Consequently, we categorized all predicted chromatin interactions into two classes, namely, “within-TAD” and “between-TAD” (Fig. 4A). For the reference TADs derived by ArrowHead, Chrombus predictions classify the chromatin interactions within- and between TADs with an area under the receiver operating characteristic (AUROC) of 0.832, compared to that of the true Hi-C scores, 0.928 (Fig. 4D). As for HiCexplorer-derived TADs, the AUROC values are 0.861 and 0.766 for Hi-C and Chrombus, respectively (Fig. 4D). In both cases, Chrombus predictions are capable to distinguish chromatin interactions within and between TADs with noninferiority to original Hi-C data (*P* < .05). We further computed the TAD-separation score based on binarized Chrombus prediction (Supplementary Methods), and our data showed high consistency between truth and prediction (Pearson correlation coefficient = 0.725, *P* < 2.2 × 10^−16^, Fig. 4B and C).

3D chromatin enables interactions among different *cis*-elements, which underlie the function of eQTL. Hence, we validated Chrombus predictions for known eSNP–eGENE interactions and enhancer–gene interactions in GM12878 from the published database [53–55]. For comparison, we also used the true Hi-C scores to validate these interactions. As a result, in pairs of CTCF-segments encompassed known eSNP–eGENE interactions and enhancer–gene interactions, the predicted scores by Chrombus are significantly higher than the background (*P* < .05, Fig. 4E). A similar tendency can be observed in Hi-C scores. Furthermore, we found that the fold-of-enrichment of both classes of interactions increases with the Chrombus predicted scores as well as the Hi-C scores (Fig. 4F).

Bringing together, these data showed that Chrombus predictions not only approximate the known chromatin 3D structure from a fine-grained perspective but also recapitulate highly relevant *cis-*regulatory events.

### Chrombus is generalizable across different cell lineage and species

As the chromatin features are highly lineage-specific, most models require training data and prediction in the same cell type. To test whether Chrombus can resolve the complex mechanism underlying the 3D genome, we evaluated Chrombus’ generalizability across six cell lines (GM12878, K562, MR90, HeLa-S3, HCT116, and CH12) and demonstrated Chrombus’ capability of extracting conserved regulatory programs of 3D-genome. In each round of the test, the best model based on one of the cell lines was selected and validated in the other cell lines for predictive performance (Supplementary Figs 10 and 11).

**Figure 5:**
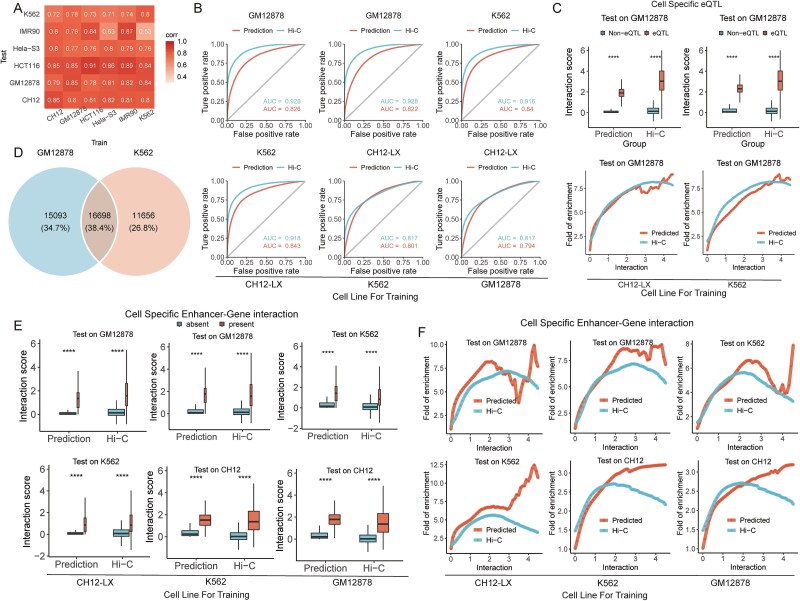
Chrombus is generalizable among different cell linage and species. (A) Pearson correlation coefficients between Hi-C scores and Chrombus prediction scores from 5-fold cross-validation crossing six cell lines, GM12878, K562, IMR90, HeLa-S3, HCT116, and CH12. For each round of validation, the model parameters are trained by one cell line and tested by another. (B) ROC curves for cross-cell-line classification of within- and between-TAD chromatin interactions using predicted scores and the true Hi-C scores by models trained on different cell lines. The predictive power of Chrombus retained in different cell lines. (C) Comparison of the interaction scores by Chrombus and Hi-C for segment pairs encompassing eQTL interaction and those do not in GM12878 cell line (upper) and fold-of-enrichment for known eQTL–eGENE pairs corresponding to different thresholding of the predicted scores (lower). In both cases, the model parameters were trained by data from a different cell line. (D) Identifying the enhancer–gene interactions that are specific to the GM12878 and K562 cell lines. These interactions are used for the analysis in (E) and (F). (E) The scores based on Chrombus prediction and Hi-C for CTCF-segment pairs encompassing known enhancer–gene interactions and those that do not. (F) Fold-of-enrichment for known enhancer–gene interactions corresponding to different thresholding of the predicted scores. In all cases, the predictions for each given cell line were based on a model in which the parameters were trained by a different one.

As a result, we observed the model trained on human cell lines is capable of predicting chromatin interactions in mouse-derived cell lines (CH12) (PCC = 0.8 ~ 0.82), and a trivial decrease in the prediction performance when trained on CH12 (PCC = 0.72 ~ 0.85) (Fig. 5A). HCT116 emerged as the most well predicted cell line (PCC = 0.84 ~ 0.89), and K562 as the least (PCC = 0.71 ~ 0.78). The model trained on GM12878 demonstrated the most stable cross-cell line predictive power (PCC = 0.77 ~ 0.85). Our findings suggest that Chrombus is capable of capturing conserved latent biological signals from highly variable chromatin features independent of the tissue types or species and thereby resolving 3D chromatin structure in more diverse biological contexts.

We further evaluated the generalizability of Chrombus by recapitulating TAD in cell lines that were not used as training data (Supplementary Methods). In all cases, Chrombus trained on one cell line was capable of inferring chromatin interactions in the other two different cell lines and recapitulates the TAD structure (Fig. 5B). In addition, Chrombus was able to distinguish cell-specific eQTL and enhancer–gene interactions across different test cell lines (Fig. 5C–E). For instance, using a model trained on GM12878, we were able to accurately predict known eQTL interactions and enhancer–gene interactions in CH12 and K562, respectively, consistent with the trends observed in the true Hi-C scores. We also noticed that the enrichment of eQTL and enhancer–gene interaction increases with Chrombus prediction scores regardless of the training set (Fig. 5F). When we performed the same analyses using the baseline models, Chrombus demonstrated consistently enhanced sensitivity in capturing chromatin interactions (Supplementary Figs 12–22).

These data reaffirmed that Chrombus prediction aligns with Hi-C scores and displays promising generalization capabilities, making it a useful tool in resolving chromatin interactions across different biological contexts.

### Chrombus is capable of predicting long-range chromatin interaction

Recent studies described several methods predicting 3D-genome using deep learning [30]. However, these methods are unable to predict long-range interactions over 2 Mb. Here, we compared the predictive performances of Chrombus with a baseline model and two state-of-the-art methods, Epiphany and C. Origami (Supplementary Methods).

**Figure 6:**
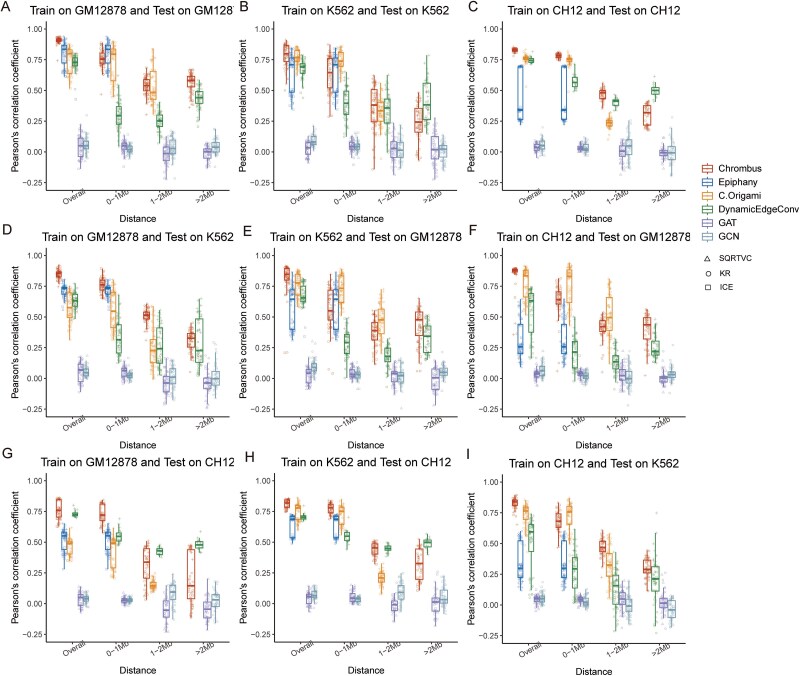
Comparison of the predictive performance for chromatin interactions of different ranges by Chrombus, epiphany, C. Origami, and baseline model: DynamicEdgeConv, GAT, GCN. (A–C) The correlation between prediction scores of chromatin interactions and the true Hi-C score for all interactions (overall), and interactions within 1 Mb, between 1 and 2 Mb, and over 2 Mb, respectively. The models were trained and tested on GM12878 (A), K562 (B), and CH12 (C). (D–I) The correlation between prediction scores and the true Hi-C scores by the models trained on one cell line and tested on another, including: trained on GM12878 and tested on K562 (D), trained on K562 and tested on GM12878 (E), trained on CH12 and tested on GM12878 (F), trained on GM12878 and tested on CH12 (G), trained on K562 and tested on CH12 (H), trained on CH12 and tested on K562 (H). In all cases, Chrombus showed comparable predictive power for chromatin interactions over 2 Mb.

As Chrombus, Epiphany, and C. Origami used differently preprocessed Hi-C data as targets, we compared the predictive performance of all methods crossing three normalization methods, Iterative Correction and Eigenvector decomposition (ICE) [56], KR [57], and SQRTVC [7] (Supplementary Methods). As a result, Chrombus significantly outperformed the other methods in overall prediction correlation, regardless of the normalization methods (*P* < 2.2 × 10^−16^, Fig. 6A). We further compared the prediction performance of the three methods along with baseline model, “DynamicEdgeConv,” “GAT,” and “GCN,” for interactions in three intervals (within 1 Mb, 1–2 Mb, and over 2 Mb). In general, our data showed that the prediction correlation of all methods decreases with the distance of chromatin interaction (Tables 1–3). For chromatin interactions within 1 Mb, all the methods showed comparable performances. Epiphany (Median PCC = 0.834) performed better in GM12878. Then, C. Origami (Median PCC = 0.738) and Chrombus (Median PCC = 0.782) showed the best performance in K562 and CH12, respectively. As for interactions within 1–2 Mb, Chrombus (Median PCC = 0.354–0.540) manifested better performance than C. Origami (Median PCC = 0.238–0.483) and “DynamicEdgeConv” (Median PCC = 0.252–0.413) in all three cell lines. Moreover, Chrombus was able to predict interactions over 2 Mb with relatively stable performance (Median PCC = 0.243–0.572) as observed for 1–2 Mb, which gives Chrombus the unique advantage of predicting long-range chromatin interactions. In addition, although “DynamicEdgeConv” exhibited inferior performance in predicting interactions <1 Mb, it surpassed C. Origami and Epiphany for interactions over 1 Mb (Fig. 6A–C). These findings reaffirmed the unique advantage of Chrombus in predicting long-range chromatin interactions.

**Table 1:** Median Pearson correlation coefficient between Hi-C scores and the prediction scores from epiphany, C. Origami, DynamicEdgeConv, GAT, GCN, and Chrombus. All models were trained and tested on the GM12878 cell line. Chrombus outperforms the other methods in predicting interactions at distances of 1–2 Mb and beyond 2 Mb.

**Methods**	0–1 M	1–2 M	>2 M	Overall
**Epiphany**	**0.834**	–	–	0.834
**C.Origami**	0.795	0.483	–	0.798
**DynamicEdgeConv**	0.294	0.252	0.442	0.728
**GAT**	0.050	−0.014	0.000	0.050
**GCN**	0.019	0.028	0.039	0.050
**Chrombus**	0.755	**0.540**	**0.582**	**0.908**

The bold values indicate the best predictive performance obtained from the corresponding methods and the dash symbols mean the corresponding methods are not applicable in those ranges.

**Table 2:** Median Pearson correlation coefficient between Hi-C scores and predicted scores from epiphany, C. Origami, DynamicEdgeConv, GAT, GCN, and Chrombus. All models were trained and tested on the K562 cell line. Chrombus outperforms the other methods in predicting interactions at distances of 1–2 Mb.

**Methods**	**0–1 M**	**1–2 M**	**>2 M**	**Overall**
**Epiphany**	0.710	–	–	0.710
**C.Origami**	**0.738**	0.334	–	0.767
**DynamicEdgeConv**	0.395	0.357	**0.383**	0.691
**GAT**	0.044	0.028	0.019	0.033
**GCN**	0.043	0.014	0.023	0.081
**Chrombus**	0.645	**0.381**	0.243	**0.799**

The bold values indicate the best predictive performance obtained from the corresponding methods and the dash symbols mean the corresponding methods are not applicable in those ranges.

**Table 3:** Median Pearson correlation coefficient between Hi-C scores and predicted scores from epiphany, C. Origami, DynamicEdgeConv, GAT, GCN, and Chrombus. All models were trained and tested on the CH12 cell line. Chrombus outperforms the other methods in predicting interactions at distances of 0–1 Mb.

**Methods**	**0–1 M**	**1–2 M**	**>2 M**	**Overall**
**Epiphany**	0.342	–	–	0.342
**C.Origami**	0.755	0.238	–	0.763
**DynamicEdgeConv**	0.565	**0.413**	**0.501**	0.744
**GAT**	0.030	0.005	−0.006	0.037
**GCN**	0.023	0.049	−0.008	0.052
**Chrombus**	**0.782**	0.354	0.31	**0.780**

The bold values indicate the best predictive performance obtained from the corresponding methods the dash symbols mean the corresponding methods are not applicable in those ranges.

We also compared the generalizability of the six methods by inferring 3D chromatin structures of the test cell lines after training by data from a different one (Fig. 6D–I, Supplementary Methods). For models trained on GM12878 and tested on K562, our data showed that the performance of Chrombus is highly stable for interactions up to 2 Mb compared to the other methods, of which the prediction correlation dropped by 0.068–0.452 (Fig. 6D and I). Nevertheless, for interactions over 2 Mb in K562, although Chrombus outperformed the other methods, the median prediction PCC also dropped by 0.063–0.541. We also inferred 3D chromatin structures of GM12878 (Fig. 6E and F) and CH12 (Fig. 6G and H) in the same way, and Chrombus showed relatively better performance in predicting chromatin interactions than other methods.

To investigate the distance-dependent sensitivity and stability of models, we systematically partitioned the distance of chromatin interactions into 100 kb genomic intervals. Our analysis revealed that the correlation between Chrombus’ prediction and true Hi-C score increased progressively with genomic distance, demonstrating the advantage of Chrombus in predicting long-range interactions (Supplementary Fig. 23). To ascertain that the performance of Chrombus is not dependent on particular genomic coordinates, we first replaced the input coordinates by positioning encoding based on rank-based fragment identifiers. As a result, the predictive performance was retained. Then, we removed positional information completely from the input features, resulting in 40% performance decline (Supplementary Fig. 25).

It is also worth noting that the normalization methods used to process the training data also influence the predictive performance of these methods. All methods work better with SQRTVC than KR and ICE in GM12878 dataset normalization data (Supplementary Fig. 24). However, we found Chrombus the least sensitive to the normalization methods in predicting the CH12 cell line (Supplementary Fig. 24E and G–I).

## Discussion

Resolving the 3D genome offers great advantages to the understanding of the *cis*-regulation of gene expression in eukaryotes. Although many experimental methods have been developed to depict local or genome-wide landscape of chromatin interactions, retrieving the contact map in the context of heterogeneous tissues or diseases remains challenging. Recent advances in deep learning yielded many successes in solving the dynamics of biomolecules, such as protein 3D structure and ligand–receptor docking [58, 59]. These methodologies provide an alternative approach to *in silico* prediction of the 3D conformation of the genome based on more accessible features, such as DNA sequences and epigenetic marks. Indeed, several deep-learning methods have demonstrated their potential to predict DNA loops [60–63].

The organization of 3D-genome involves complex biological processes. DNA loop extrusion by cohesion, anchored by CTCF, represents the most common conformation elements of 3D-genome [64]. Thus, CTCF-based DNA segment serves as a better basic unit of chromatin interaction than evenly distributed bins. In addition, CTCF-based DNA segment also provides a better base for summarizing input epigenetic features, which are also unevenly distributed. Finally, CTCF-based DNA segments enable the graph representation of 3D-genome, which fits better to the topology of chromatin interactions than conventional convolution methods.

We propose a novel graph convolutional model, Chrombus-XMBD, to predict chromatin interactions using readily available and cost-effective epigenetic data. Chrombus introduces distance-based regularization on the conventional multihead attention leverages, which enable sensitive detection of the pattern of chromatin interactions. These characteristics of Chrombus make it stand out of existing methodologies and hence offer complementary predictive power to previous models. Our approach interprets chromatin interactions as a graph, which allows for extraction and aggregation of features from interacting DNA segments. A recent study demonstrates that the vast majority of chromatin loops (86%) are bound by CTCF and RAD21, and a large fraction of loops (38%) are located at contact domain boundaries [65]. Therefore, Chrombus uses CTCF binding to partition chromatin into unevenly sized DNA fragments, which serve as elementary units of interaction. Unlike other methods, Chrombus uses graph representation of 3D-genome that allows passing and aggregation of relevant information among neighboring fragments in the highly irregular spatial domains of DNA. Thus, the inferred interactions by Chrombus reveal the interactive dynamics of all CTCF-based segments, and the embeddings can inform the semantic structure of the epigenome underlying the spatial organization of chromatin.

Chromatin features are crucial for predicting the 3D-genome, but not until recently did few models predict chromatin interactions sole by chromatin features. Chrombus uses six common features: DNA accessibility, CTCF, RAD21, POLR2A, H3K4me3, and H3K27ac. Epiphany uses DNA accessibility, CTCF, H3K27ac, H3K27me3, and H3K4me3, and C. Origami uses DNA accessibility, CTCF, and DNA. As noted in previous studies [7, 65], chromatin 3D interactions exhibit a strong distance dependency, where spatially proximal genomic segments interact more frequently. Our data reaffirmed that location is the most deterministic of chromatin interactions, and replacing the current input of position information by positional encoding in natural language models, such as Bidirectional Encoder Representations from Transformers (BERT) [66], resulted in slightly improved model performance (Supplementary Fig. 25). These results reflect the importance of relative positioning or vicinity in chromatin interactions. The contributions of other features changes by the distance of segments. In total, CTCF binding, H3K27ac, H3K4me3, POLR2A binding, DNA accessibility, and CTCF motif score are more important than CTCF motif strand and RAD21 binding (Supplementary Fig. 9A and B). Among these input features, H3K4me3 and H3K27ac are commonly known as marks of promoters and enhancers; studies have shown that the landscape of both features is rather independent and highly variable in different regulatory contexts [26, 51]. POLR2A is important and essential for loop formation, and its recruitment and elongation are often facilitated by H3K27ac-marked regions, as this modification creates an open chromatin environment conducive to transcription machinery binding [67, 68]. These marks are often colocalized at regulatory elements within TADs, suggesting a coordinated mechanism for transcriptional regulation (Supplementary Fig. 33) [69, 70]. Therefore, by incorporating the input features, our model can better resolve the complex dynamics of the 3D genome and thereby predict chromatin interactions *de novo*. The importance of these chromatin features is in line with the known biology of 3D-genome. However, other new chromatin features are yet to be discovered to further improve the prediction.

In our data, long-range chromatin interactions (>2 Mb) account for 52.97% (CH12-LX)–88.30% (HCT116) of positive interactions. Yet, the maximal prediction range for most of current methods is no more than 2 Mb. Long-distance interactions are particularly useful to resolve large chromatin structures, explaining global reprogramming of chromatin architecture occurs during early mammalian development [71], larger difference in chromatin fiber compaction of long distance [72], long-range regulatory interactions (eQTL and trait-associated loci) [73], and the regulation of higher-level chromatin structures, including compartments, which are closely linked to the occurrence and development of diseases [1, 74, 75]. In addition, Chrombus has proved to be more generalizable, which makes it suitable for prediction of 3D-genome in cell-type and tissue level. However, we found performance differences between models trained using the K562 dataset and GM12878 dataset. The GM12878-model tends to have more robust performance than the K562-model in predicting interactions across chromosomes and cell lines. Deeper sequencing depth of GM12878 (~4 billion contacts) than K562 (~0.9 billion contacts) may determine the results. It ought to be mentioned that the generalizability of Chrombus was evaluated only on human- and mouse-derived cell lines, hence at the level of tissue or lineage. To test the generalizability of Chrombus at a more general level, further study should include more distant species.

We also observed differences in the Chrombus’ performance due to three different coverage normalization methods. SQRTVC [7] is based on simple normalization by the sum of respective rows and columns and takes the square root to supplement the over-correction. ICE iterates the normalization process until all bins of contact matrix are equally visible, whereas KR derives an optimized scaling vector to achieve the same goal. Our data suggest that all three methods’ best predictive performances are conditioned upon the Hi-C normalization methods, suggesting that the training of these methods are affected by the technical biases introduced by preprocessing of data.

Nevertheless, Chrombus-XMBD is limited by the quality training data. The availability and quality of the chromatin features are highly variable among different batches, platforms, and sample types. Owing to its generalizability, Chrombus can be readily applied to training data of missing features or different noise structure by transfer learning.

On the other hand, several factors may contribute to the drop of the predictive performance of Chrombus in certain cell lines. First of all, there is still a possibility that the parameter set picks unrelated biases nested in the specific training data; then, the low quality and low resolution of some datasets can affect the training efficiency. Finally, the preprocessing steps such as the normalization method can also introduce extra biases in the training data.

Moreover, the experimental technique and the analytical pipeline for Hi-C is not fully matured [27]. The training and validation of Chrombus is subject to unknown biases or noises. Currently, we use TAD inferred from Hi-C, eQTLs, and enhancer–gene interactions to validate Chrombus predictions but the most straightforward evidence should base on experimental results such as 3C and 4C.

In summary, we described Chrombus-XMBD, a graph model predicts 3D genome *ab initio*. The unique advantages of Chrombus greatly facilitate the exploration of the intricate regulatory mechanisms underlying 3D chromatin structure and the consequential impacts on transcription.

**Key Points**

- We developed “Chrombus-XMBD,” a graph convolution model for predicting 3D chromatin interactions based on common epigenetic features.
- “Chrombus-XMBD” outperforms existing algorithms in efficiently predicting long-range chromatin interactions.
- “Chrombus-XMBD” showcases remarkable generalizability across human and mouse-derived cell lines.

## Supplementary Material

supplementary_information_20250331_bbaf183

## Data Availability

The training and validation of “Chrombus” are based on data of six cell lines, GM12878, K562, MR90, HeLa-S3, HCT116, and CH12 (Supplementary Tables 3–5). All data analyzed in this study were published previously [65, 76, 77]. The code for training and predicting with Chrombus, and trained model are available at https://github.com/bioinfoheroes/Chrombus-XMBD.
