# Supplementary material for: Chrombus-XMBD: a graph convolution model predicting 3D-genome from chromatin features
Source: Brief Bioinform. 2025 May 2;26(3):bbaf183. doi: 10.1093/bib/bbaf183 (PMC12047703; doi:10.1093/bib/bbaf183)
Supplement: supplementary_information_20250331_bbaf183 [file supplementary_information_20250331_bbaf183.docx]

# Supplementary Information

## Supplementary methods

## Graph representation of 3D-genome based on CTCF-segments

We segmented the chromatin into uneven fragments based on CTCF-binding peaks. Each fragment corresponds to a vertex, denoted as $\boldsymbol{V}_{\boldsymbol{i}}\boldsymbol{\in}\boldsymbol{V}$ (Supplementary Table 2). Then the edge, $\boldsymbol{E}_{\boldsymbol{ij}}$, between the vertices *i* and *j* were defined by the averaged interaction score between the two fragments derived directly from processed Hi-C data**.** Thus, the 3D-structure of the chromatin is represented by a graph $\boldsymbol{G}\mathbf{(}\boldsymbol{V}\mathbf{,}\boldsymbol{E}\mathbf{)}$ (Figure 1). Each vertex is represented by 14-dimension feature vector$\boldsymbol{x}_{\boldsymbol{i}}$ . The features include CTCF-binding strength at both ends of the segment, denotes as left peak and right peak. Then we inferred the directionality of CTCF motifs within the CTCF-binding peaks using fimo. Each CTCF-peak was labeled as “0”, “1” according to the positive and negative strand. The RAD21-binding at a given CTCF-site (“left cohesin” and “right cohesin”) was also processed as a binary status, where “1” indicated that RAD21-binding peak was in vicinity of the CTCF-site (within a 500bp).

Furthermore, we incorporated mean value of H3K27ac, H3K4me3, POLR2A and DNase-I signals to represent the epigenetic character of each segment, which were scaled to the range of [0, 1]. Finally, we included the chromosomal coordinates of both ends of a segment, resulting in a total of 14 node attributes (Supplementary Table 2, Supplementary Figure 26-31). As the edges in the graph depends on the strength of interaction, we filtered out edges of weak interaction (raw Hi-C value > 0). To define the edge attributes or, the interaction potential of the segment pairs, we calculated the mean contact values of all 5kb-bin or 10kb-bin located in each segment. There are three methods provided by JuicerTools for normalizing the contact values: SQRTVC, KR and VC. To determine the most appropriate normalization method, we chose the normalization method which best discriminate interactions within the same TAD (“within-TAD”) and those spanning different TADs (“between-TAD”) by cross-entropy (Supplementary Figure 32AB). A higher cross-entropy indicates a better discriminative power between the two groups. The TADs were inferred from normalized Hi-C data using the Arrowhead algorithm. Based on our evaluation, SQRTVC was chosen as normalization method (Supplementary Figure 29C). We also computed the mean value of epigenetic signals within two segments. We observed that the segment-pairs in “within-TAD” showed very slightly higher signal for POLR2A and H3K27ac than in “between TAD”, but no difference for the other two marks (Supplementary Figure 33).

All the features we use were preprocessed by ENCODE and 4DN standard pipeline. We downloaded “fold change over control (bigwig)” and “bed-narrowPeak”. for CTCF ChIP-seq data, “bed-narrowPeak” for RAD21-ChIP-seq data, “fold change over control (bigwig)” for H3K27ac, H3K4me3, POLR2A and DNase I (Supplementary Table 3-4).

## The Chrombus Model

We developed “Chrombus”, a generative graph convolution model which reconstructs chromatin interaction (Figure 1). Chrombus adopts a typical GAE model. The encoder maps 14-dimensional features of a graph of *N* vertices $\boldsymbol{x=(}x_{1}\boldsymbol{, \ldots,}x_{N}\boldsymbol{)}$ to 32-dimensional representation$\boldsymbol{z}=(z_{i},\ldots,z_{N})$ through 3-depth dynamic edge-convolution layers. And layer 3 is stepped over a single nonlinear layer from to layer 1. Given $\boldsymbol{z}$, the decoder then generates *N*-by-*N* matrix of interaction strength as $\boldsymbol{A}=\boldsymbol{z}\boldsymbol{'}\boldsymbol{z}$ , for each pair of vertices.

The graph convolution layers follow an architecture of stacked self-attention, dynamic edge convolution and fully connected layers for encoder (Figure 1).

The encoder takes batched subgraphs as input. Each subgraph contains 128 vertices, representing 128 consecutive CTCF-segments which were randomly cropped from a chromatin. During training and testing, the vertices were connected by random edges based on following rules. 1) no interactions beyond the maximal span of 32 consecutive CTCF-segments; and 2) within the maximal span, any pair of CTCF-segments are connected by 50% chance, which correspond to an Erdős-Rényi random graph of G (128, 0.5).

The encoder consists three edge-convolution layers, each of which transform the node features from the previous layer by incorporating messages from random assigned neighbors. The output dimensions of each layer are 32, 16, 32, respectively (Figure 1). Let normalized features of the target node *i* in the first layer $h_{i}^{(0)}$ be the transformed from input feature $x_{i}^{\left( 0 \right)}$ and features of the neighboring source nodes $j\in N(i)$ (eq. 1). Subsequently, the hidden variable of the $l$-th layer, $h_{i}^{(l)} (\forall i\in\{1,2,...,128\})$ is obtained by $l$-th $(\forall l\in\{1,2,3\})$graph convolution operator:

| $h_{i}^{(0)}=W^{0}\cdot\left[ {x_{j}^{\left( 0 \right)}-x_{i}^{\left( 0 \right)}\vert\vert x}_{i}^{\left( 0 \right)} \right]+ b^{0}$  $h_{i}^{l+1}=\sigma(\sum_{j\in N\left( i \right)} a_{ij}^{l}{[W}^{l}\cdot{[h}_{j}^{l}-h_{i}^{l}\vert\left\vert h_{i}^{l} \right]+b^{l}])$ | (1) |
| --- | --- |

Where $W^{l}$ and $b^{l}$ denotes the trainable weights and bias of the $l$-th layer, $\sigma$ represents the sigmoid activation function, $N(i)$ represents the neighbors of node $i$, $i$ and *j* represent the indices of target and source nodes, respectively. $a_{ij}^{l}$ is partition matrix that gathers and reallocates the weight of hidden representations from $N(i)$ in the $l$-th layer.

To ensure the target node receives relevant information from its random neighbors during edge-convolution, we employed a multi-head (n=8) self-attention as described in transformer model (eq. 2). We used three 1-depth linear layers to determine query, key and value. The attention score $a_{ij}^{l}$ is defined as follow:

| $Query=W^{q}[h_{j}^{l}-h_{i}^{l}\vert\vert h_{j}^{l}]$  $Key=W^{k}[h_{j}^{l}-h_{i}^{l}\vert\vert h_{j}^{l}]$  $Value=W^{v}[h_{j}^{l}-h_{i}^{l}\vert\vert h_{j}^{l}]$  $a_{ij}^{l}=\frac{exp\{\sigma(W_{a}^{l}(Query\cdot Key))\}}{\sum_{N(i)} exp\{\sigma(W_{a}^{l}(Query\cdot Key))\}}$ | (2) |
| --- | --- |

Where $W_{a}^{l}$represents the weights of attention, and $[\cdot||\cdot]$ denotes the concatenation of multiple vectors. The increment of hidden expression of node *j* over its center node *i* is passed through the edge when calculating$a_{ij}^{l}$.

In addition to self-attention, we design a signed edge weight to address the biological fact that interaction between segments is negatively correlated with the linear distance (eq. 3). We defined a *cis*-interaction range of 9 consecutive segments so that the sign of $\omega$ flips according to $D_{ij}$, the linear distance between two segments measured by number of segments in between.

| $\omega_{ij}=log(\frac{D_{ij}{\vert Index}_{i}- {Index}_{j}\vert}{9})$ | (3) |
| --- | --- |

**Decoder**

To acquire the interaction scores among nodes, we apply inner production to the aforementioned embedding of each pair of all nodes, which is as follow:

|  | $Interaction Score= \prod_{i=1}^{N} \prod_{j=1}^{N} h_{i}^{T}h_{j}$ | (4) |
| --- | --- | --- |

Where the $Interaction Score$ represents the interaction strength inferred by decoder between node i and node j. The mean square error (MSE) is broadly used for regression problems. Eventually, our objective of model is to minimize MSE between predicted and real interaction of target nodes.

## Model training and testing

We trained the model using two distinct approaches. Firstly, to predict the whole genome across chromosomes, we employed a “leave-one-out” model on GM12878. The dataset was randomly put-back cropped into 128-segments from one chromosome as testing chromosome, and the other 21 chromosomes were used for training. For model training, 200 samples were generated from each chromosome, and 50 samples for testing purposes. During testing, we applied a sliding window of 128-segments on each chromosome with a step size of 9. The predicted Hi-C scores represent the average scores of the overlapping interactions. The consistency between true Hi-C score and Chrombus’ prediction is evaluated using the Pearson’s correlation coefficient, analyzed with the Pandas library. These predictions are subsequently utilized for downstream analyses, including TAD, eQTL and enhancer-gene interaction analysis.

Regarding the generalization model, chromosomes are cropped into non-overlapping samples using a 128-segment window. In total, 301 (GM12878), 420 (K562), 455 (CH12), 383 (HCT116), 325 (HeLa-S3) and 328 (IMR90) samples are generated. Out of these samples, 50 samples are randomly selected for testing. To train the model, a five-fold cross-validation approach is applied, utilizing the least number of samples. Four model are trained, and the model with the best performance on the testing set is selected for cross-cell prediction.

In our experiments, we employed an early stopping strategy with a patience value of 50 epochs. This means that training would halt if no improvement in the monitored metrics (MSE and Pearson’s correlation coefficient) was observed over 50 consecutive epochs. And the remember strategy we utilized is "RANKED", which updates the best values and resets the patience counter as long as any of the monitored metrics shows improvement, even if the other metrics remain stable or do not improve simultaneously.

## Comparing interactions located Within TADs and between TADs

For GM12878 and K562, TADs were download from GSE63525 with resolution of 5kb and 10kb. For HCT116 CH12, HeLa-S3 and IMR90, TADs are inferred by Arrowhead based on Hi-C data with resolution of 10kb. TADs inferred by HiCexplorer were based on Hi-C data of 5kb-resolution and 10kb-resolution. Interactions between two segments within a TAD were categorized as “Within TAD”, while the interactions between two segments located in two, different adjacent TADs were categorized as “Between TADs”. For TADs defined by both methods, we examined the chromatin interactions of these two groups according to the Chrombus’ predictions across 22 autosomes. To assess the ability of interaction scores to distinguishing between the two groups, we plot receiver operating characteristic (ROC) curves using R-package of ROCR, and calculated the area under the curve (AUC) values using the R-package ROCit. We performed non-inferior test to compare the predicted score to Hi-C scores using rocNIT package.

**TAD separation score**

We chose threshold of 1.4 to divide predictions interactions into groups of “Within-TAD” and “Between-TAD” according to AUROC value, then interactions of “Within-TAD” and “Between-TAD” were encoded as “0” and “1”, respectively. For each CTCF-binding site, we calculated TAD separation scores as in previously published study [1]. In our setting, the nodes represent CTCF-segments instead of evenly sampled bins, and the edges were average interactive strength. To obtain the TAD separation score, we defined the upstream 9 segments of a CTCF-binding site as left region, and downstream 9 segments of CTCF-binding site as right region, and then comparing the contact counts between the left regions and right region to the nts within such regions. Then we calculated the TAD separation scores as follows:

$$S= \frac{\sum_{i\in L;j\in R} C_{i,j}}{min(sum\left( C_{L} \right), sum(C_{R}))}$$

Where C denotes the contact counts, L and R represent the left and right region. Finally, we evaluated the correlation between TAD separation scores based on Chrombus’ predictions and those based on true Hi-C signals using Pearson’s correlation coefficients.

## Enrichment of eQTLs, enhancer-gene interactions

eQTLs of GM12878 with hg19 coordinates were download from GTEX (Version 7)[2]. Enhancer-gene interactions of GM12878, K562, HCT116, HeLa-S3 and IMR90 were obtained from GeneHancer, and CH12’s was download from EnhancerAtlas. The eQTLs and enhancer-gene interactions are referred as interaction events. We categorized each pair of segments into two groups: those encompass interaction events and those do not. We then compared the distribution of chromatin interaction scores between these two groups, and the significance of the difference was evaluated using the Wilcoxon test.

To calculated the fold of enrichment, we considered the total number of predicted segment pairs as $N$ and the total number of segment pairs encompassing interactions as $n$. The background ratio was calculated as $n/N$. Then we applied incremental thresholding to the predicted scores, for all segments with predicted scores above the threshold value, we calculated the ratio of segment pairs encompassing interaction events ($n^{'}/N^{'}$). The enrichment fold was then calculated by $(n^{'}/N^{'})/(n/N)$.

## Comparing Chrombus with other methods

In addition to SQRTVC, we replicated the process employed by Epiphany to produce contact counts using ICE and KR normalization. ICE normalization is processed by HiCExplorer. The models were retrained on GM12878 with identical labeling. Chromosomes of 6, 9, 11 and 19 were randomly chosen for training, while the remaining chromosomes served as the testing set for models, including Epiphany and C.Origami, and baseline model, including DynamicEdgeConv, GCN and GAT. Subsequently, the interactions were divided into three groups based on their distance: within 1M, between 1-2M and beyond 2M. Then at a much finer scale, we divided interactions by increasing interval of 100kb.

Within each distance group, we calculated the Pearson correlation coefficient between the Hi-C scores and predicted scores from all models. All the calculations and visualizations were performed using R (version 4.1.2).

## Segment clustering

We randomly selected 100 segments from each of the 22 autosomes in the GM12878 cell line, yielding a total of 2,200 segments for analysis. The features of each segment are represented by the 32-dimensional embeddings obtained from the Chrombus and adjusted by dividing by distance. We applied Kernel PCA dimensionality reduction to the features of segments using “kpca” function from the “kernellab” package[3], with the “kernel” parameter set to "rbfdot". The top two principal components are selected based on the explained variance for segment clustering. With a resolution parameter set to 0.3, we identified two clusters, named “type-1” and “type-2”. Clustering is performed using the Louvain method, implemented in the cluster_louvain function from the “igraph” package[4].

## Rank-based position encoding

We employed "rank-based positional encodings" to replace the absolute positions of chromatin segments. This involves assigning a sequential number to each of the 128 segments in a subgraph based on their chromosomal order, ranging from 1 to 128. The "order-based positional encodings" were then converted into positional embeddings.

## Ablation experiment of positional encoding

We modified the input features of the Chrombus model by removing the positional encoding, reducing the feature dimension from 14 to 12. To assess the effect of this modification, we compared the training process, including MSE loss, and the Pearson’s correlation coefficient between the predicted and true values in both the training set and testing set. The model was trained with an early stopping strategy, where the patience parameter was set to 50 and the maximum number of epochs was 500.

## Supplementary Table 1-5

Supplementary Table 1 Model parameters of Chrombus

| **Parameter** | **Value** |
| --- | --- |
| cis_span | 3, 6, 9, 12, 15, 18, 21 |
| max_span | 32, 64, 128 |
| n_head | 4, 8 |

Supplementary Table 2 Preprocessing of 14-dimensional node features

| Raw data | Preprocessing | Feature | Raw value | Normalization | Processed value |
| --- | --- | --- | --- | --- | --- |
| CTCF ChIP-Seq | fimo | Strand of 3’ CTCF motif | Discrete value (“+” / “-”) from fimo | Convert “+” to 1 and “-” to 0 | 1 / 0 |
|  |  | Scores of 3’ CTCF motif | Continuous numeric value (> 0) from fimo | Min-max | Range (0, 1) |
|  |  | Strand of 5’ CTCF motif | Discrete value (“+” / “-”) from fimo | Convert “+” to 1 and “-” to 0 | 1 / 0 |
|  |  | Scores of 5’ CTCF motif | Continuous numeric value (> 0) from fimo | Min-max | Range (0, 1) |
| CTCF ChIP-Seq | fold-change-over-control | 3’ CTCF binding peak signal | Continuous numeric value (> 0) | Min-max | Range (0, 1) |
|  |  | 5’ CTCF binding peak signal | Continuous numeric value (> 0) | Min-max | Range (0, 1) |
| Histone ChIP-Seq |  | H3K27ac signal | Continuous numeric value (> 0) | Min-max | Range (0, 1) |
|  |  | H3K4me3 signal | Continuous numeric value (> 0) | Min-max | Range (0, 1) |
| TF ChIP-Seq |  | POLR2A signal | Continuous numeric value (> 0) | Min-max | Range (0, 1) |
| DNase-Seq |  | DNase I signal | Continuous numeric value (> 0) | Min-max | Range (0, 1) |
| TF ChIP-Seq | bed-narrowPeak | RAD21 binding (left) | Boolean | Convert “True” to 1 and “False” to 0 | 1 / 0 |
|  |  | RAD21 binding (right) | Boolean | Convert “True” to 1 and “False” to 0 | 1 / 0 |
| Hg19 genome building |  | Start position of segment | Integer (> 0) | Position / length of chromosome | Range (0, 1) |
|  |  | End position of segment | Integer (> 0) | Position / length of chromosome | Range (0, 1) |

Supplementary Table 3 Data source (hg19) for Chrombus training

| **Data Type** | **Mark** | **File format** | **GM12878** | **CH12** | **K562** |
| --- | --- | --- | --- | --- | --- |
| CTCF | Domain Boundary | bed-narrowPeak  fold-change-over-control | ENCFF473RXY  ENCFF886KRA | ENCFF739OMJ  ENCFF025UEN | ENCFF738TKN  ENCFF933ZLL |
| RAD21 | Cohesin | bed-narrowPeak | ENCFF001VFE | ENCFF048HTU | ENCFF002CXU |
| POLR2A | RNA polymerase II mark | fold-change-over-control | ENCFF368HBX | ENCFF441BMN | ENCFF647MSS |
| H3K4me3 | Activating mark | fold-change-over-control | ENCFF818GNV | ENCFF426ODS | ENCFF291SWG |
| H3K27ac | Enhancer mark | fold-change-over-control | ENCFF180LKW | ENCFF666CND | ENCFF010PHG |
| DNA accessibility | Open chromatin signal | read-depth normalized signal | ENCFF901GZH | ENCFF228LWM | ENCFF352SET |
| Chromatin interaction | Contact counts | Contact matrix and fastq | GSE63525 | GSE63525 | GSE63525 |

Supplementary Table 4 Data source (hg38) for comparison of model’s performance

| **Data Type** | **Mark** | **File format** | **GM12878** | **K562** |
| --- | --- | --- | --- | --- |
| CTCF | Domain | bed-narrowPeak | ENCFF951PEM | ENCFF660GHM |
| CTCF | Boundary | fold-change-over-control | ENCFF644EEX | ENCFF682MFJ |
| RAD21 | Cohesin | bed-narrowPeak | ENCFF834GOT | ENCFF258VXX |
| POLR2A | RNA polymerase II mark | fold-change-over-control | ENCFF203NVD | ENCFF806LCJ |
| H3K4me3 | Activating mark | fold-change-over-control | ENCFF287HAO | ENCFF911JVK |
| H3K27ac | Enhancer mark | fold-change-over-control | ENCFF469WVA | ENCFF849TDM |
| DNA accessibility | Open chromatin signal | read-depth normalized signal | ENCFF960FMM | ENCFF972GVB |
| Chromatin interaction | Contact counts | Contact matrix and fastq | 4DNFI1UEG1HD | 4DNFITUOMFUQ |

Supplementary Table 5 Data source (hg38) for Chrombus training

| **Data Type** | **Mark** | **File format** | **HCT116** | **HeLa-S3** | **IMR90** |
| --- | --- | --- | --- | --- | --- |
| CTCF | Domain | bed-narrowPeak | ENCFF171SNH | ENCFF111RWV | ENCFF307XFM |
| CTCF | Boundary | fold-change-over-control | ENCFF620LDT | ENCFF179RSE | ENCFF256DUS |
| RAD21 | Cohesin | bed-narrowPeak | ENCFF391AAM | ENCFF239FBO | ENCFF699YDJ |
| POLR2A | RNA polymerase II mark | fold-change-over-control | ENCFF802CGI | ENCFF180XTE | ENCFF851ZRC |
| H3K4me3 | Activating mark | fold-change-over-control | ENCFF176SFX | ENCFF489CIY | ENCFF903WOV |
| H3K27ac | Enhancer mark | fold-change-over-control | ENCFF329BPA | ENCFF194XTD | ENCFF663RRL |
| DNA accessibility | Open chromatin signal | read-depth normalized signal | ENCFF110HOX | ENCFF757GHL | ENCFF971HXR |
| Chromatin interaction | Contact counts | Contact matrix and fastq | ENCFF750AOC | 4DNFIM2BMJ33 | ENCFF188SSH |

## Supplementary Figure 1-33


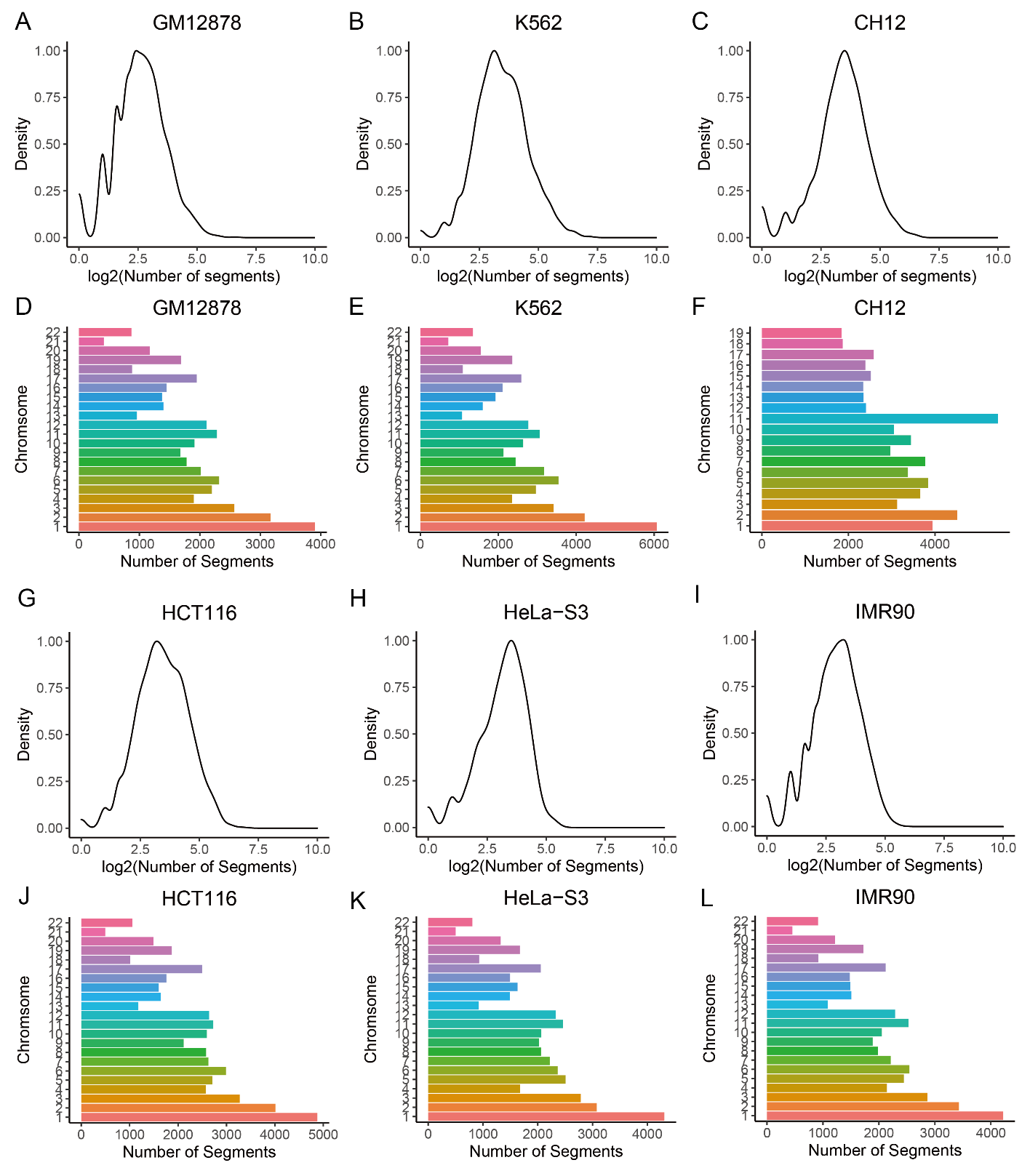


Supplementary Figure 1 Distribution of the node features of six cell lines. A-C: Distribution of the number of CTCF-segments located in one single TAD in GM12878, K562 and CH12. D-F: Segment counts by autosomes in in GM12878, K562 and CH12. G-I: Distribution of the number of CTCF-segments located in one single TAD in HCT116, HeLa-S3 and IMR90. J-L: Segment counts by autosomes in HCT116, HeLa-S3 and IMR90.


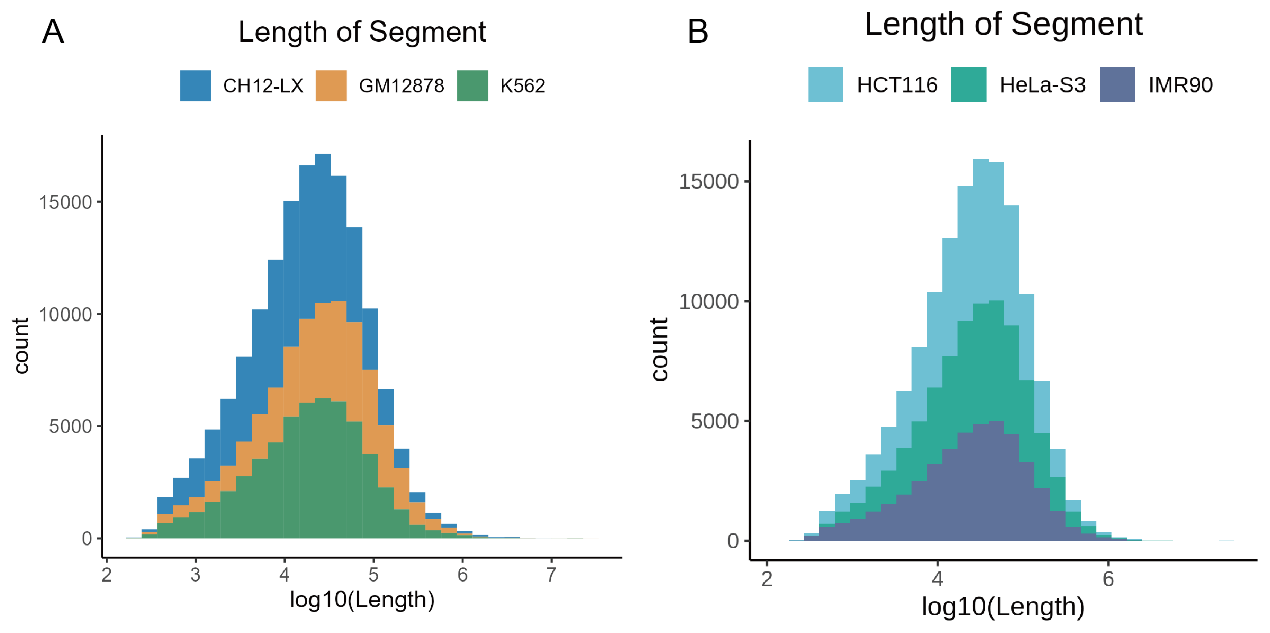


Supplementary Figure 2 Distribution of segment lengths in six cell lines. A. Segment length of GM12878, K562 and CH12 cell lines. B. Segment length of HCT116, HeLa-S3 and IMR90 cell lines.


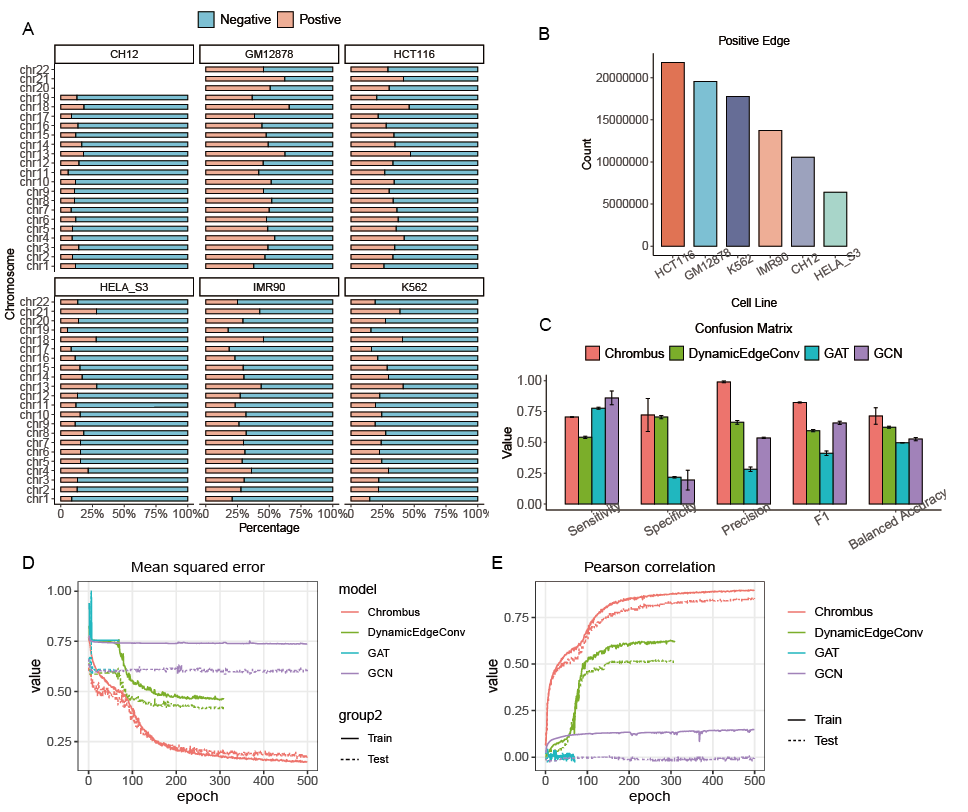


Supplementary Figure 3 Fraction of positive interactions in the training datasets (segment-pairs beyond the maximal span were excluded) of Chrombus and comparison of Chrombus versus three baseline models. A. Fraction of positive interactions for each autosome in six cell lines. B. Counts of positive interactions in six cell lines. C. The sensitivity, specificity, precision, F1, accuracy for binary statuses of chromatin interactions predicted by Chrombus performed better than the other three baseline models. D-E: Training loss and Pearson correlation coefficient of all four models up to 500 epochs.


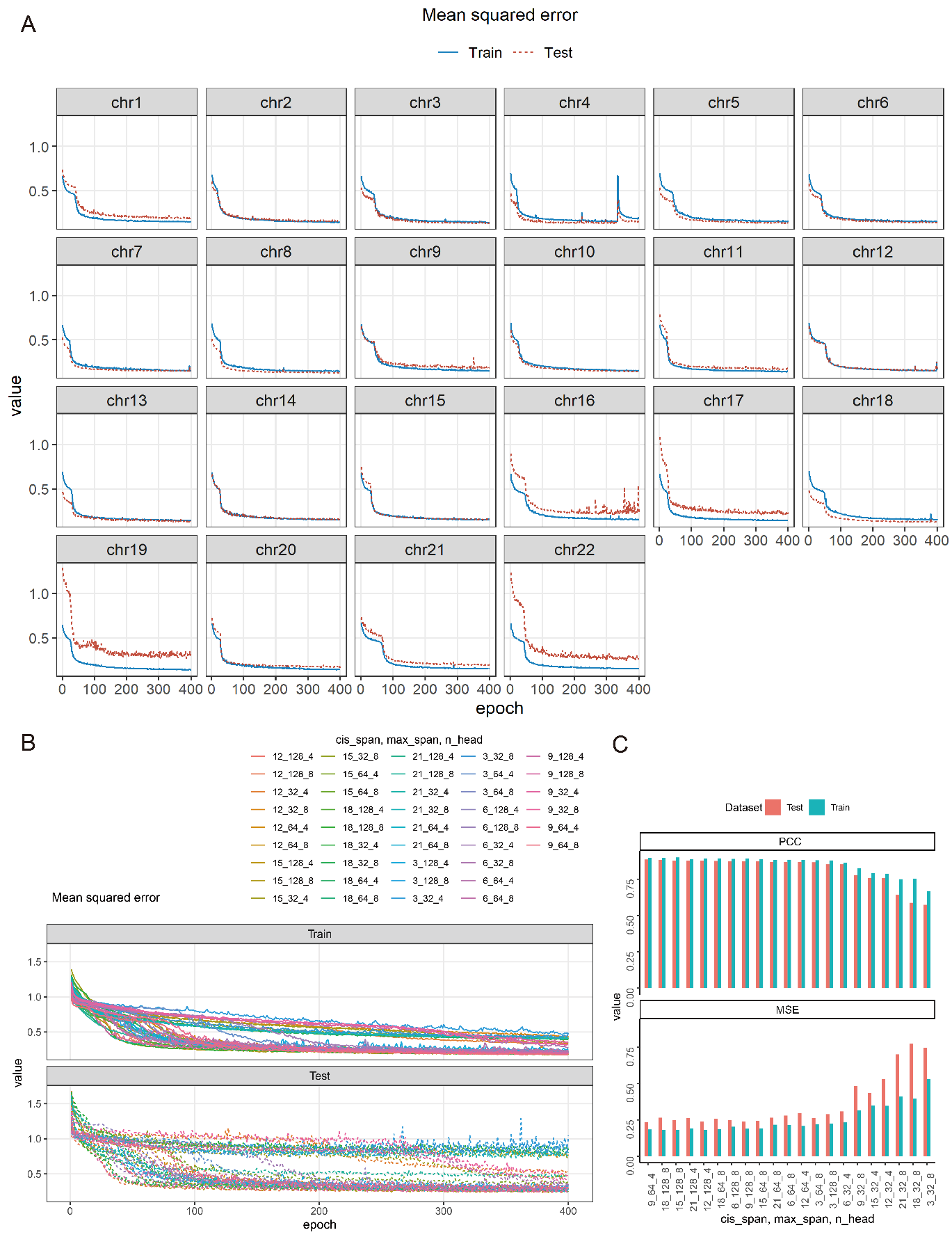


Supplementary Figure 4 Training process and model performances. A: Training and testing loss (MSE) of GM12878 model during 400 epochs. For each training process, one of 22 autosomes was left out as test data with the rest as training data. The training-test process was repeated 22 rounds until each autosome was tested independently based on model trained on the others, which resulted in 22 models. B: Parameter optimization for cis_span, max_span and n_head based on grid search. Training and testing loss during 400 epochs for each parameter setting (Supplementary Table 4). C: Performance of each parameter combination were evaluated using Pearson correlation coefficient (PCC) and MSE loss, showing the best combination of parameters (cis_span = 9, max_span = 64, n_head = 4).


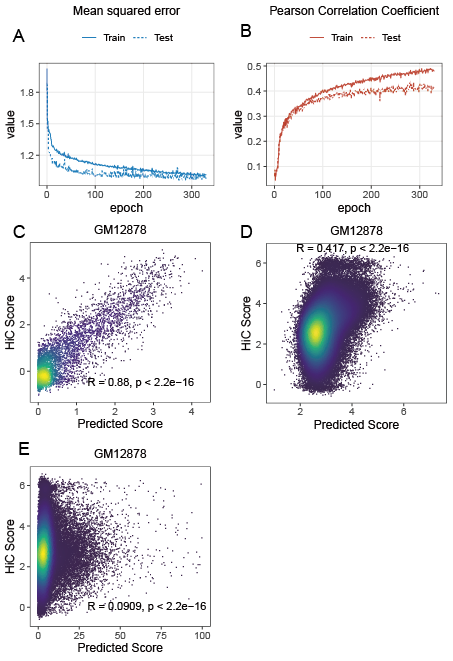


Supplementary Figure 5 Training process and predicted interaction of 10kb segments of Chrombus on GM12878. A-B. Training and testing loss (MSE) and Pearson correlation coefficient during 500 epochs, we use Early Stop to avoid overfit of model. C. Scatter plot of predicted values against true Hi-C scores of CTCF-based segments below 10kb. D. Scatter plot of predicted values against true Hi-C scores of 10kb genome bin. E. Scatter plot of predicted values against true Hi-C scores of 10kb genome bin, and the model was trained using CTCF-peak-based segmentation.


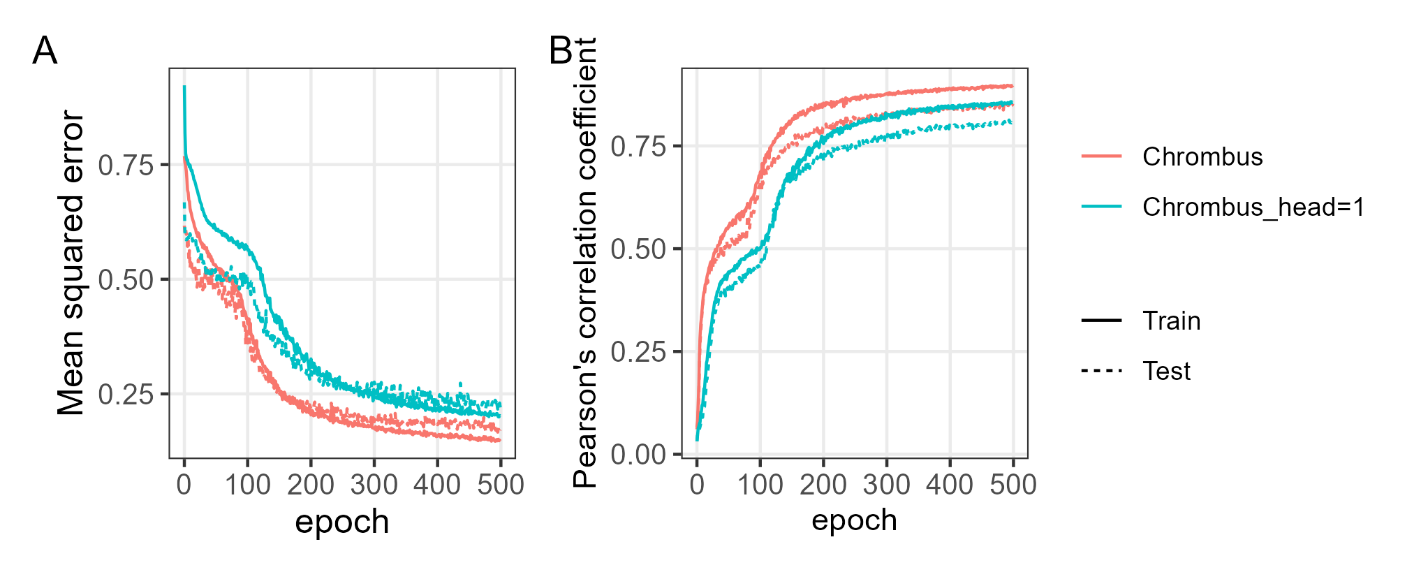


Supplementary Figure 6 Comparison of Chrombus (head = 8) versus Chrombus with 1 head. A-B: Training loss and Pearson correlation coefficient of two models during maximum of 500 epochs.


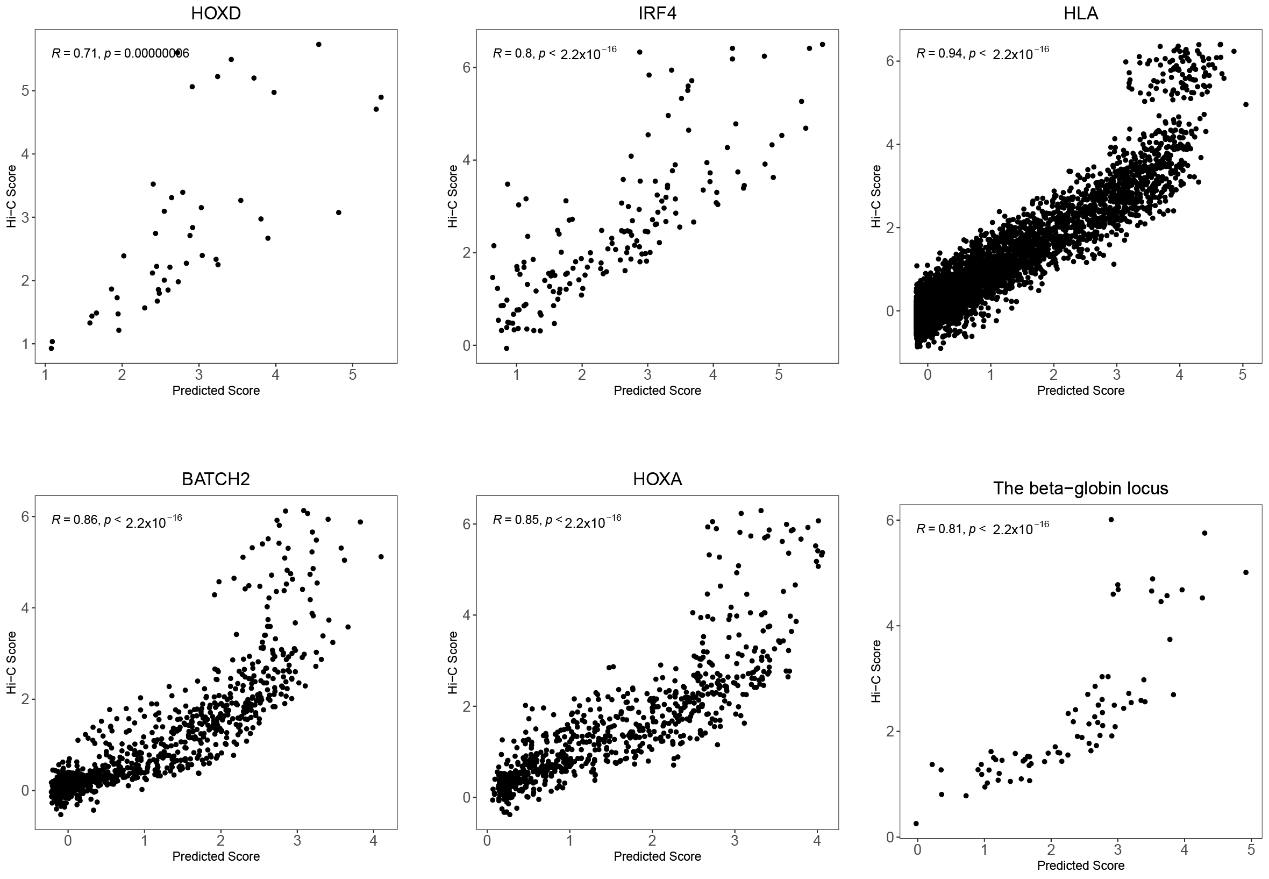


Supplementary Figure 7 Predictive performance of Chrombus for six known TAD in GM12878. The scatter plots showed the correlation between predicted scores and the true Hi-C scores (processed by SQRTVC normalization).


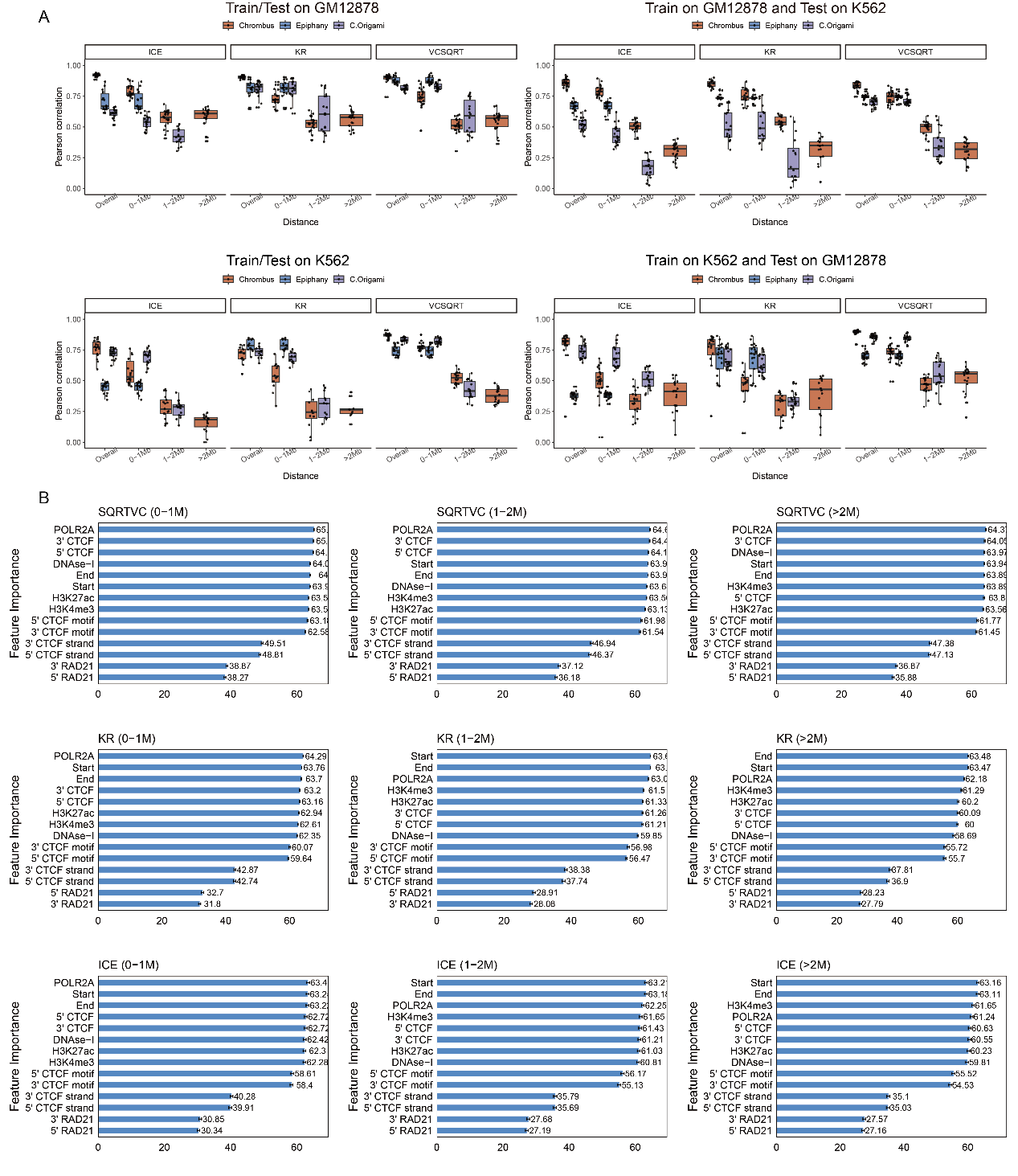


Supplementary Figure 8 The importance of the input features of Chrombus were evaluated using GNNexplainer. The importance were evaluated in different ranges and normalization methods.


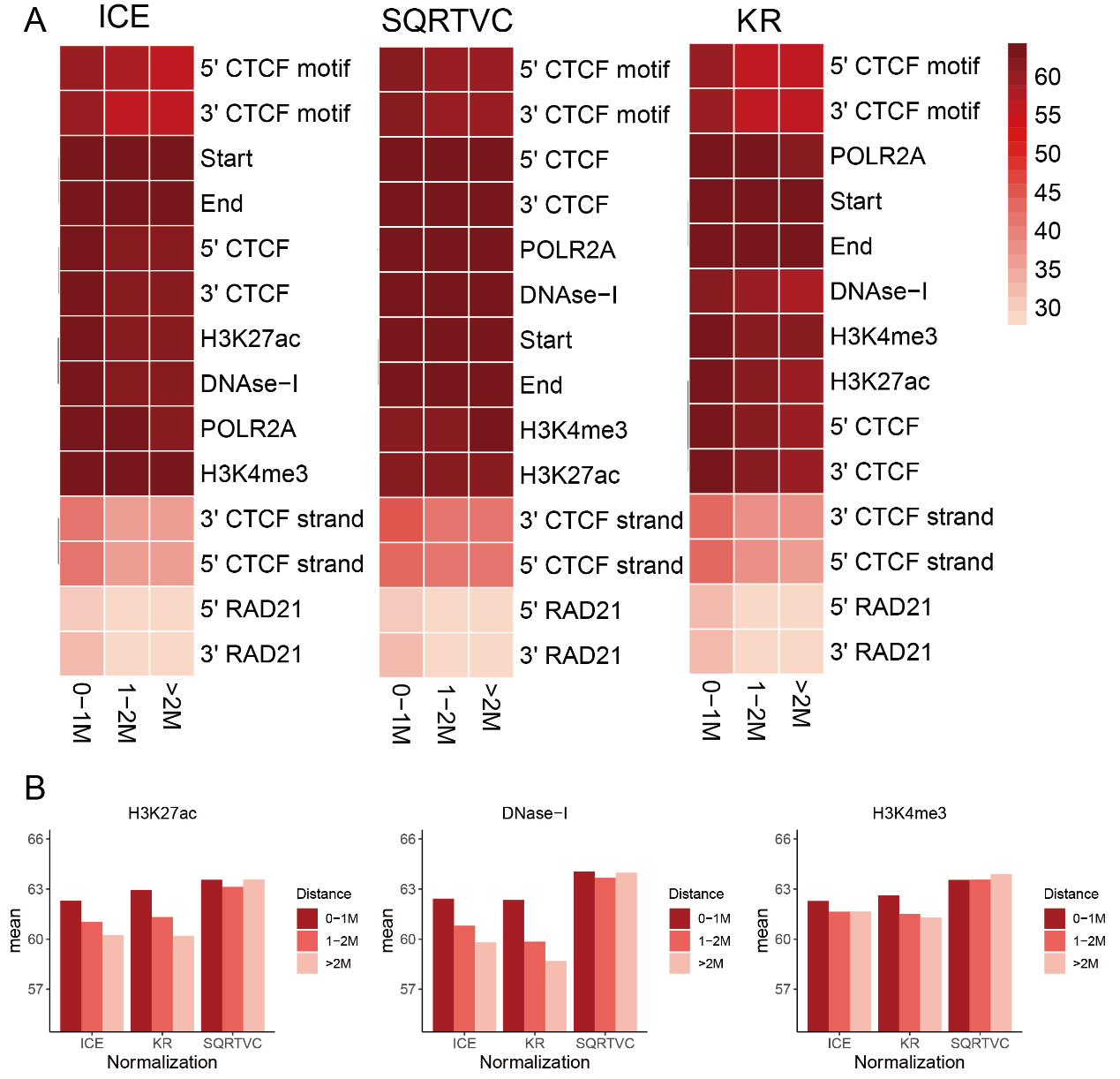


Supplementary Figure 9 The importance for all input features were evaluated using GNNexplainer. A-B: Feature importance evaluated using GNNexplainer for three different ranges and cross three normalization methods.


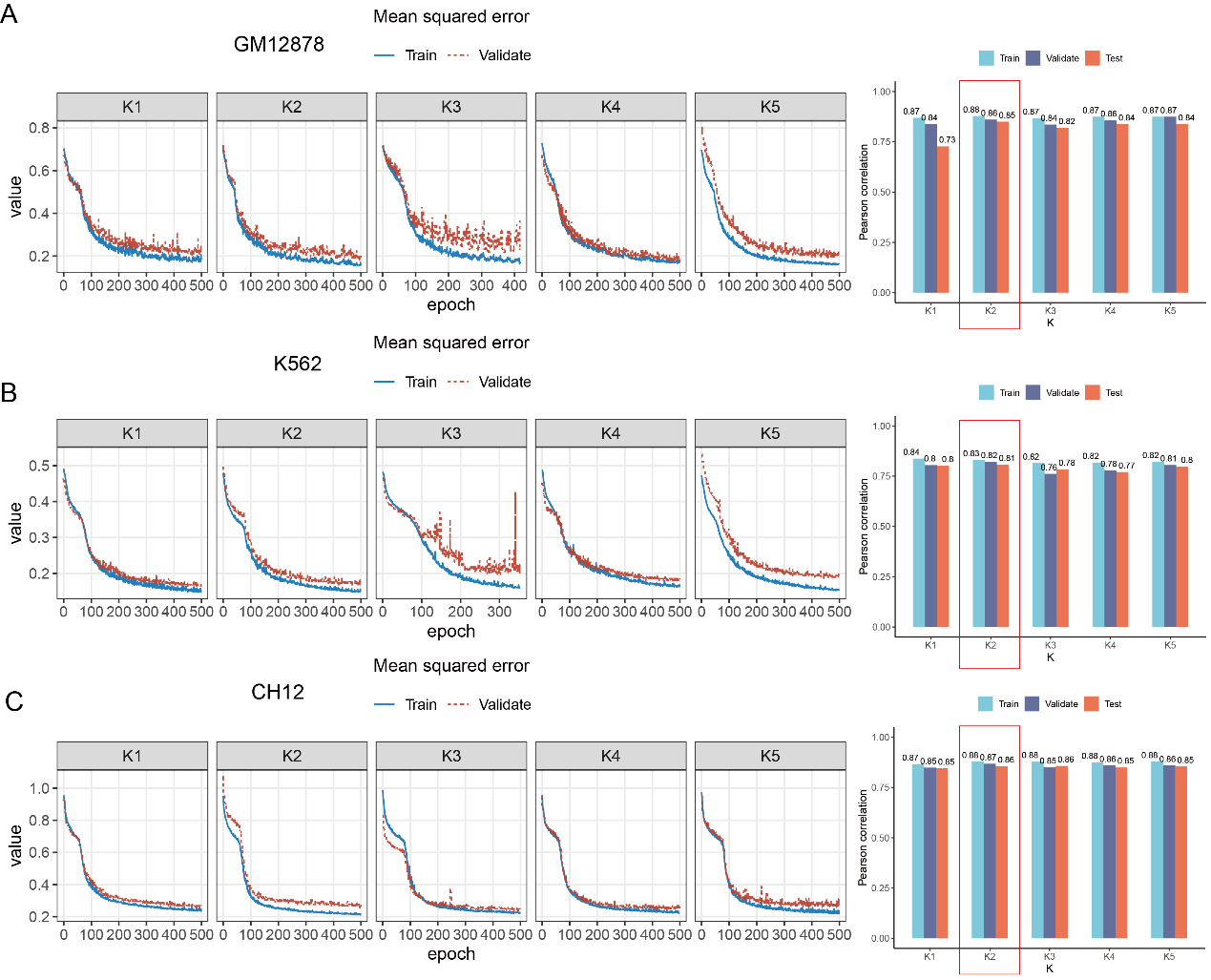


Supplementary Figure 10 Five-fold cross validation of Chrombus in three cell lines. The datasets containing subgraphs of 128 nodes were randomly divided into six parts: five for cross validation (“train” and “validation”), and one for external test (“test”). The left panels showed the training and validating loss up to 500 epochs. The right panels showed the Pearson correlation between predicted scores and the true Hi-C scores. The best model of the five was chosen by the testing results and used for cross-cell-line prediction (right, Methods). A: Validation results for GM12878. B: Validation results for K562. C: Validation results for CH12.


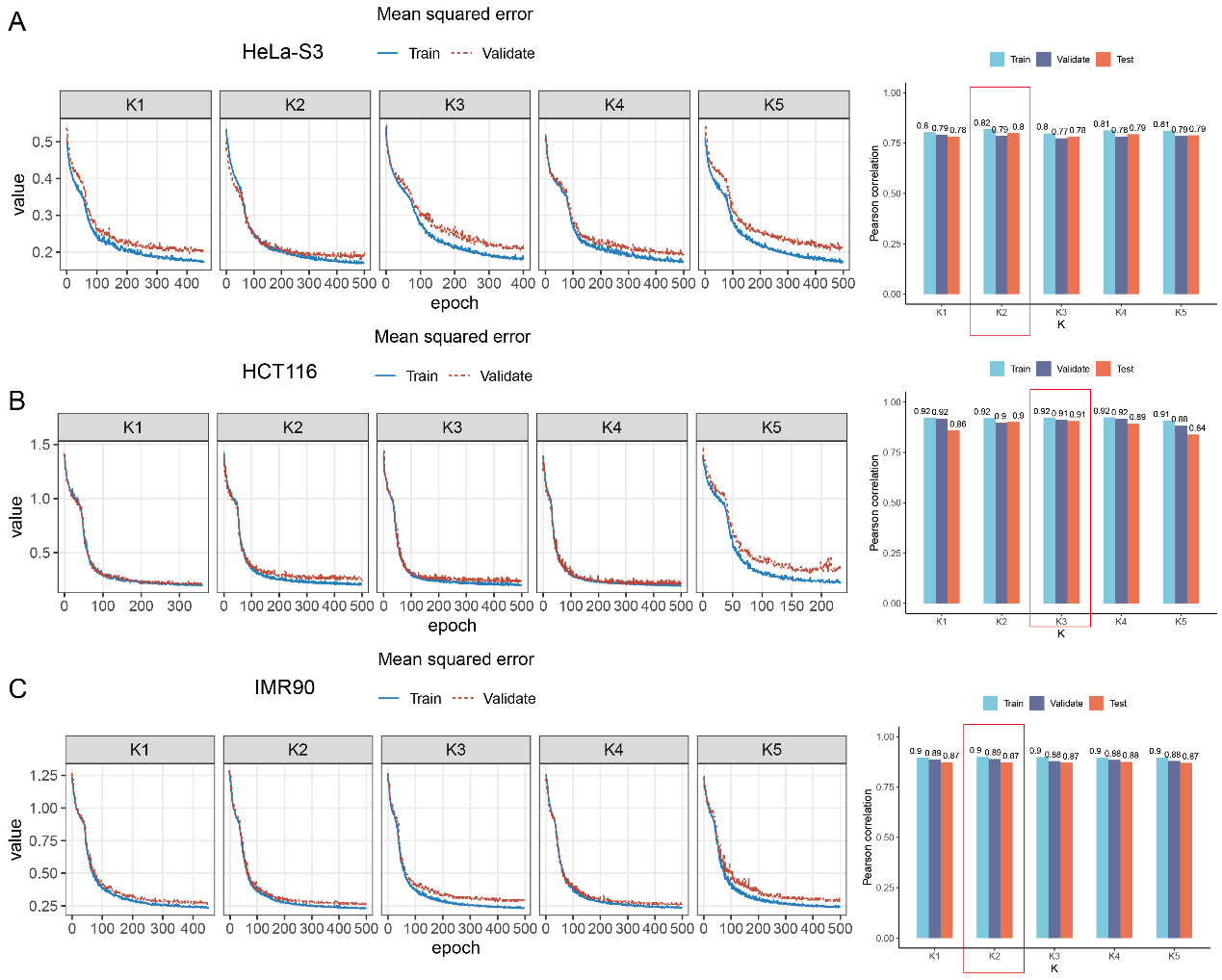


Supplementary Figure 11 Five-fold cross validation of Chrombus in three cell lines. The datasets containing subgraphs of 128 nodes were randomly divided into six parts: five for cross validation (“train” and “validation”), and one for external test (“test”). The left panels showed the training and validating loss up to 500 epochs. The right panels showed the Pearson correlation between predicted scores and the true Hi-C scores. The best model of the five was chosen by the testing results and used for cross-cell-line prediction (right, Methods). A: Validation results for HeLa-S3. B: Validation results for HCT116. C: Validation results for IMR90.


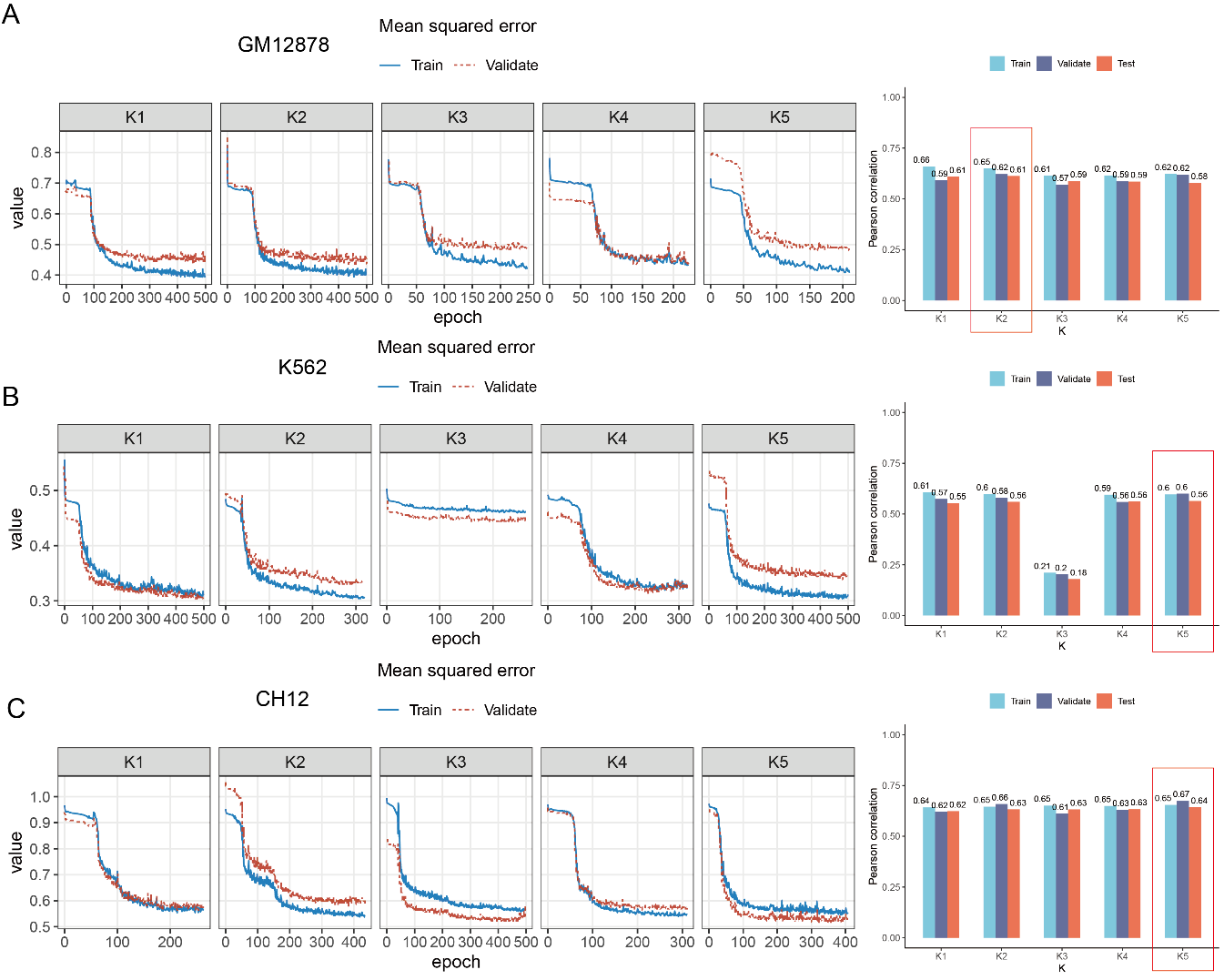


Supplementary Figure 12 Five-fold cross validation of “DynamicEdgeConv” model in three cell lines. The datasets containing subgraphs of 128 nodes were randomly divided into six parts: five for cross validation (“train” and “validation”), and one for external test (“test”). The left panels showed the training and validating loss up to 500 epochs. The right panels showed the Pearson correlation between predicted scores and the true Hi-C scores. The best model of the five was chosen by the testing results and used for cross-cell-line prediction (right, Methods). A: Validation results for GM12878. B: Validation results for K562. C: Validation results for CH12.


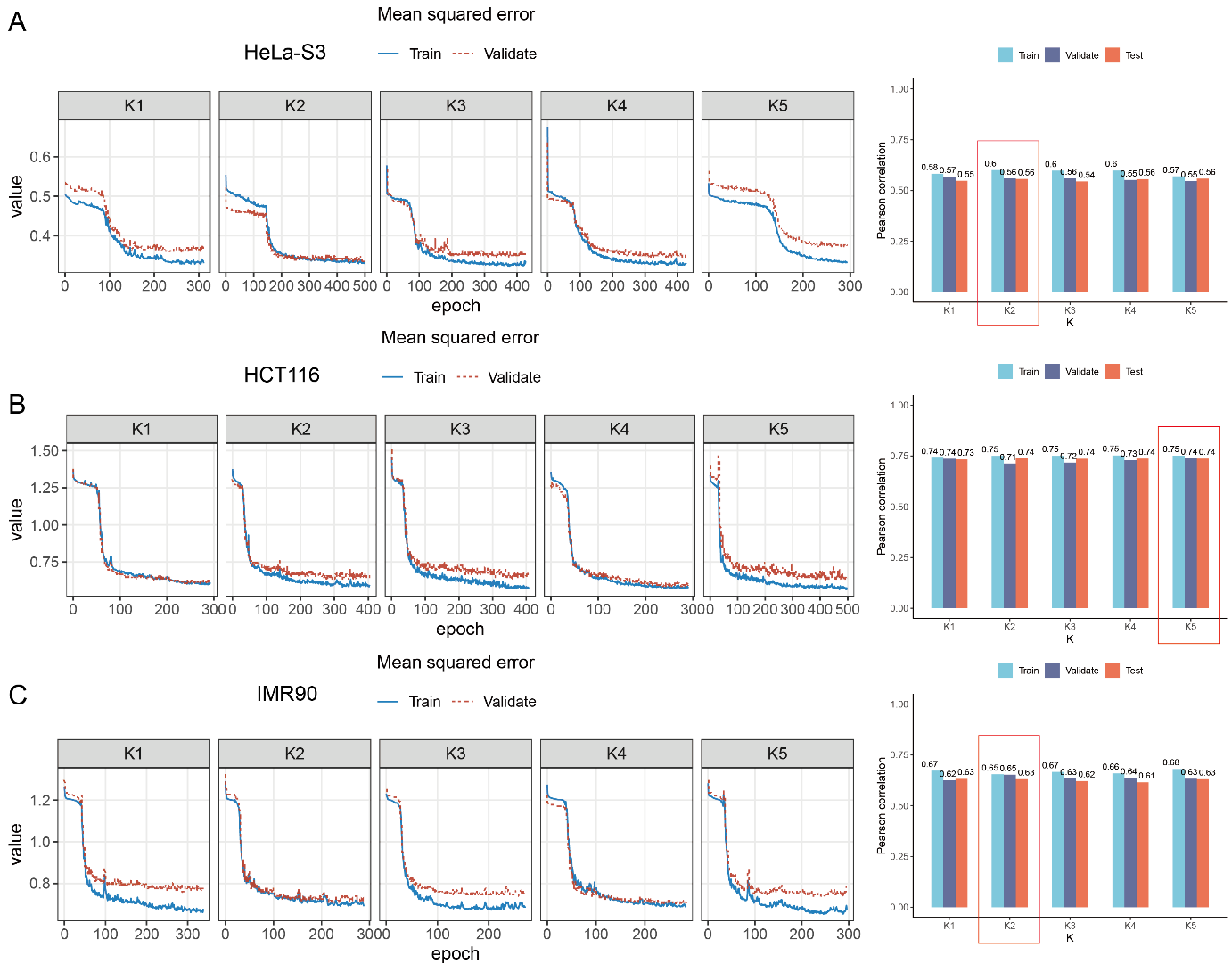


Supplementary Figure 13 Five-fold cross validation of “DynamicEdgeConv” model in three cell lines. The datasets containing subgraphs of 128 nodes were randomly divided into six parts: five for cross validation (“train” and “validation”), and one for external test (“test”). The left panels showed the training and validating loss up to 500 epochs. The right panels showed the Pearson correlation between predicted scores and the true Hi-C scores. The best model of the five was chosen by the testing results and used for cross-cell-line prediction (right, Methods). Testing of five models on test dataset and the best model was chosen for cross-cell-line prediction (Methods). A: Validation results for HeLa-S3. B: Validation results for HCT116. C: Validation results for IMR90.


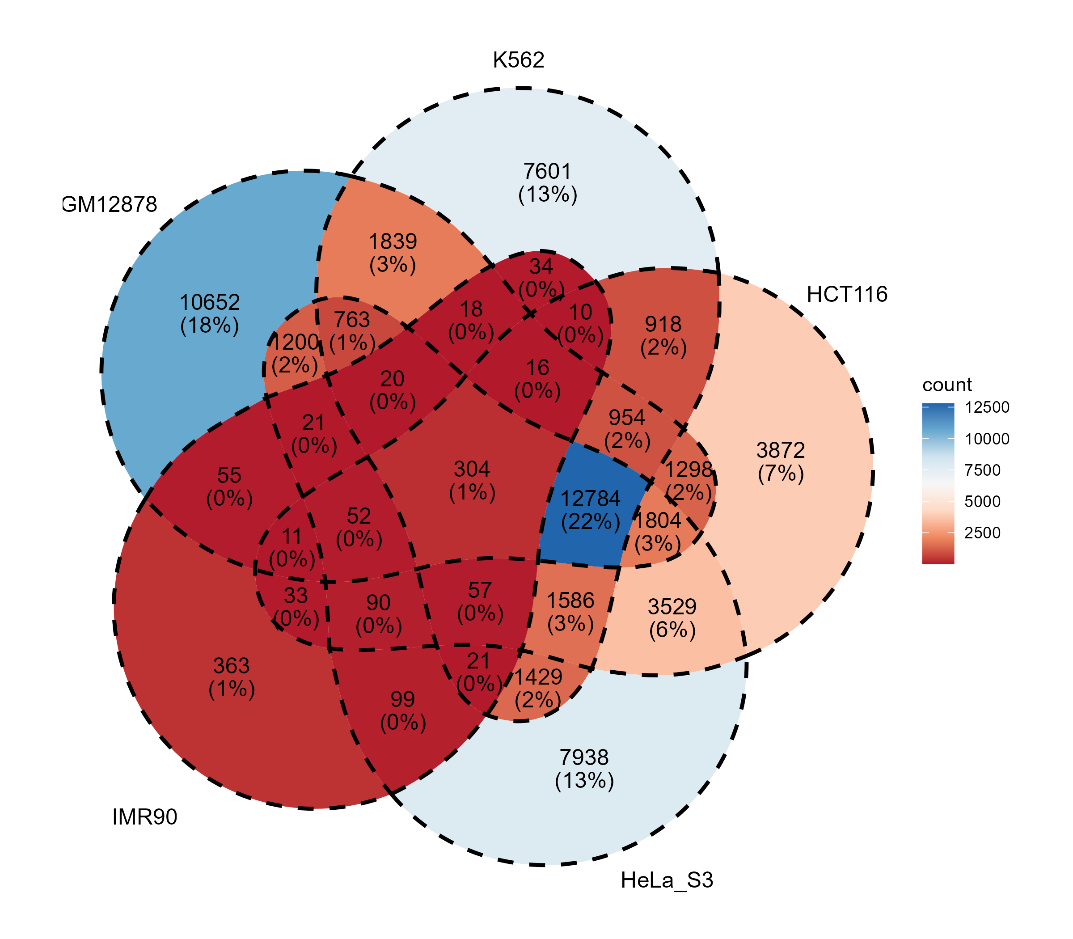


Supplementary Figure 14 Cell-specific enhancer-gene interactions in five cell lines: GM12878, K562, HCT116, HeLa-S3, IMR90. The cell-specific interactions were used for the downstream analysis.


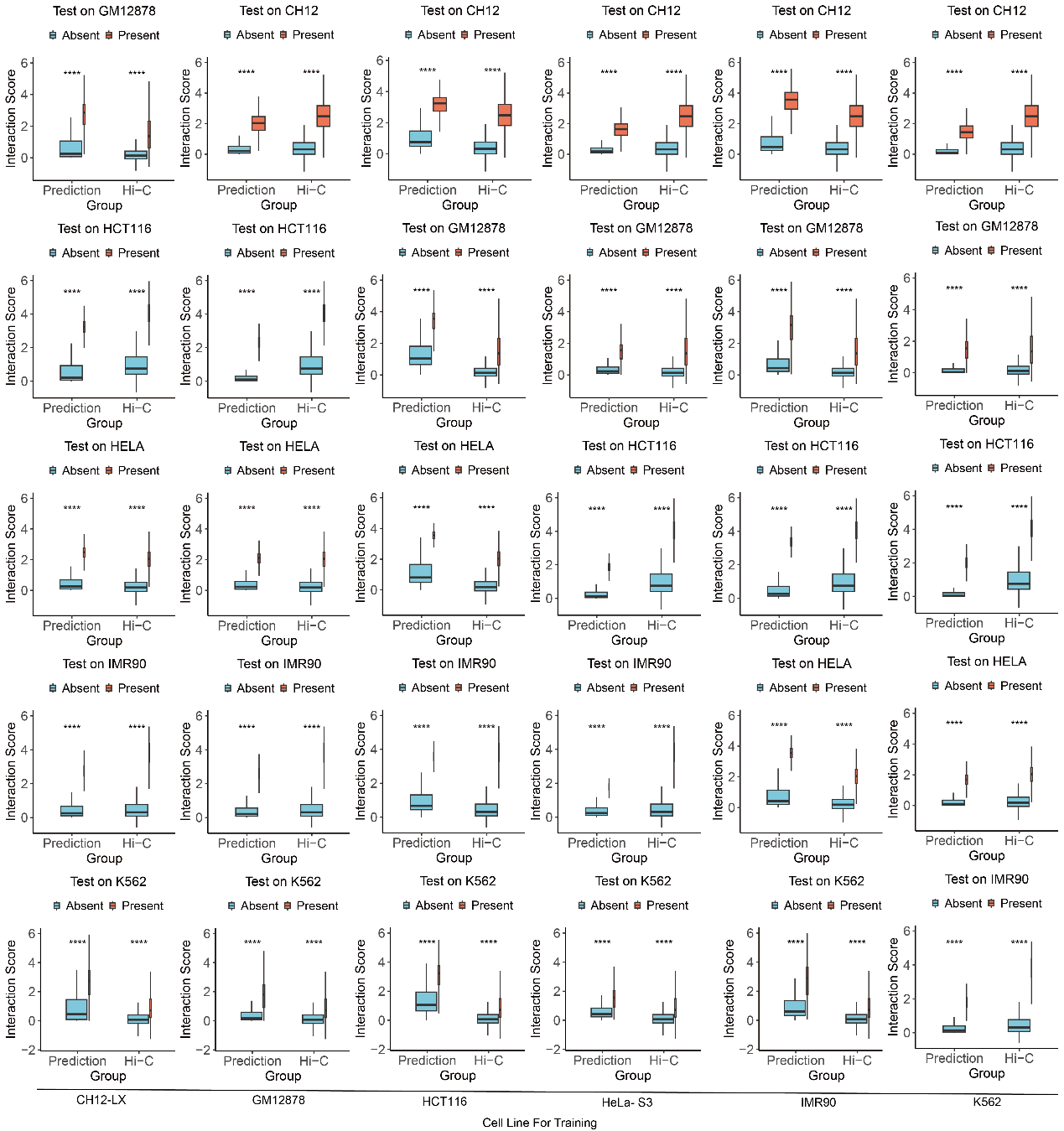


Supplementary Figure 15 The interaction scores based on Chrombus predictions and Hi-C for CTCF-segment pairs encompassing known enhancer-gene interactions and those do not. In all cases, the predictions for each given cell line were based on model of which the parameters were trained by a different one.


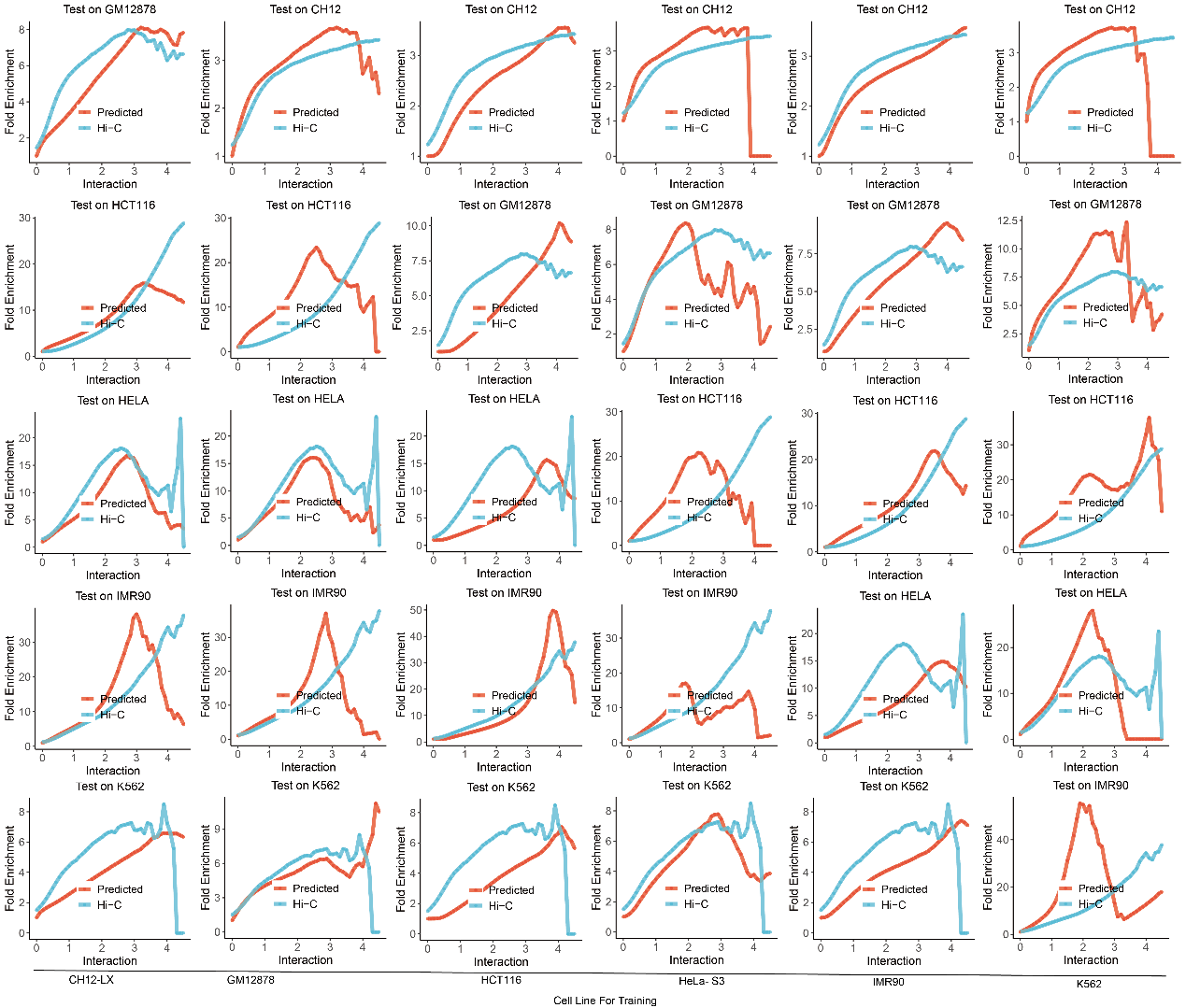


Supplementary Figure 16 The fold-of-enrichment for known enhancer-gene interactions corresponding to different thresholding of either predicted scores by Chrombus or by true Hi-C scores. In all cases, the predictions for each given cell line were based on model of which the parameters were trained by a different one.


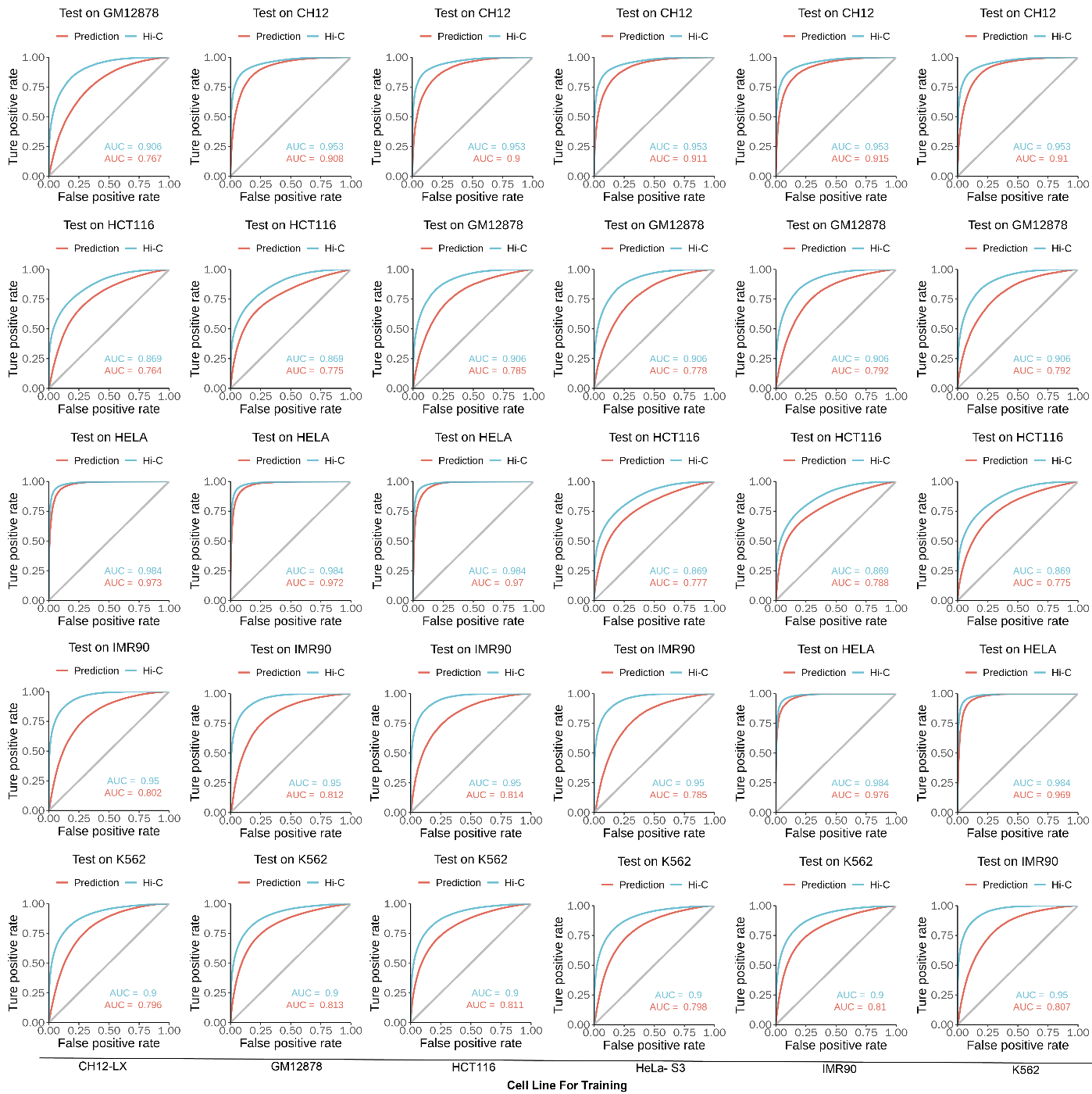


Supplementary Figure 17 The ROC curves for classification of within- and between-TAD chromatin interactions using either predicted scores by Chrombus or true Hi-C scores. The model parameters are trained using data from a different cell line. The reference TADs were inferred based on Arrowhead.


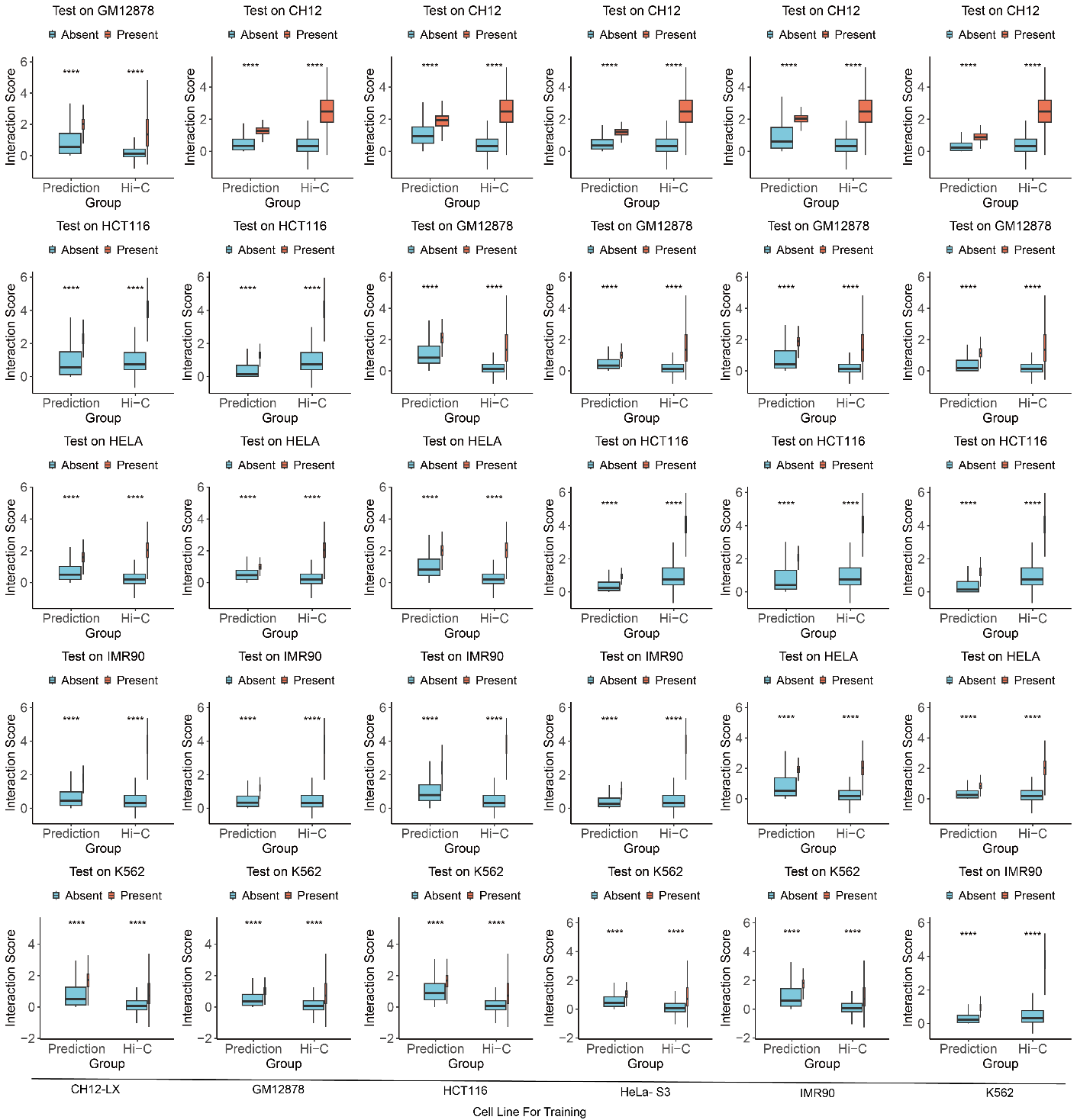


Supplementary Figure 18 The interaction scores based on either “DynamicEdgeConv” (the baseline model) prediction or true Hi-C scores for CTCF-segment pairs encompassing known enhancer-gene interactions and those do not. In all cases, the predictions for each given cell line were based on model of which the parameters were trained by a different one.


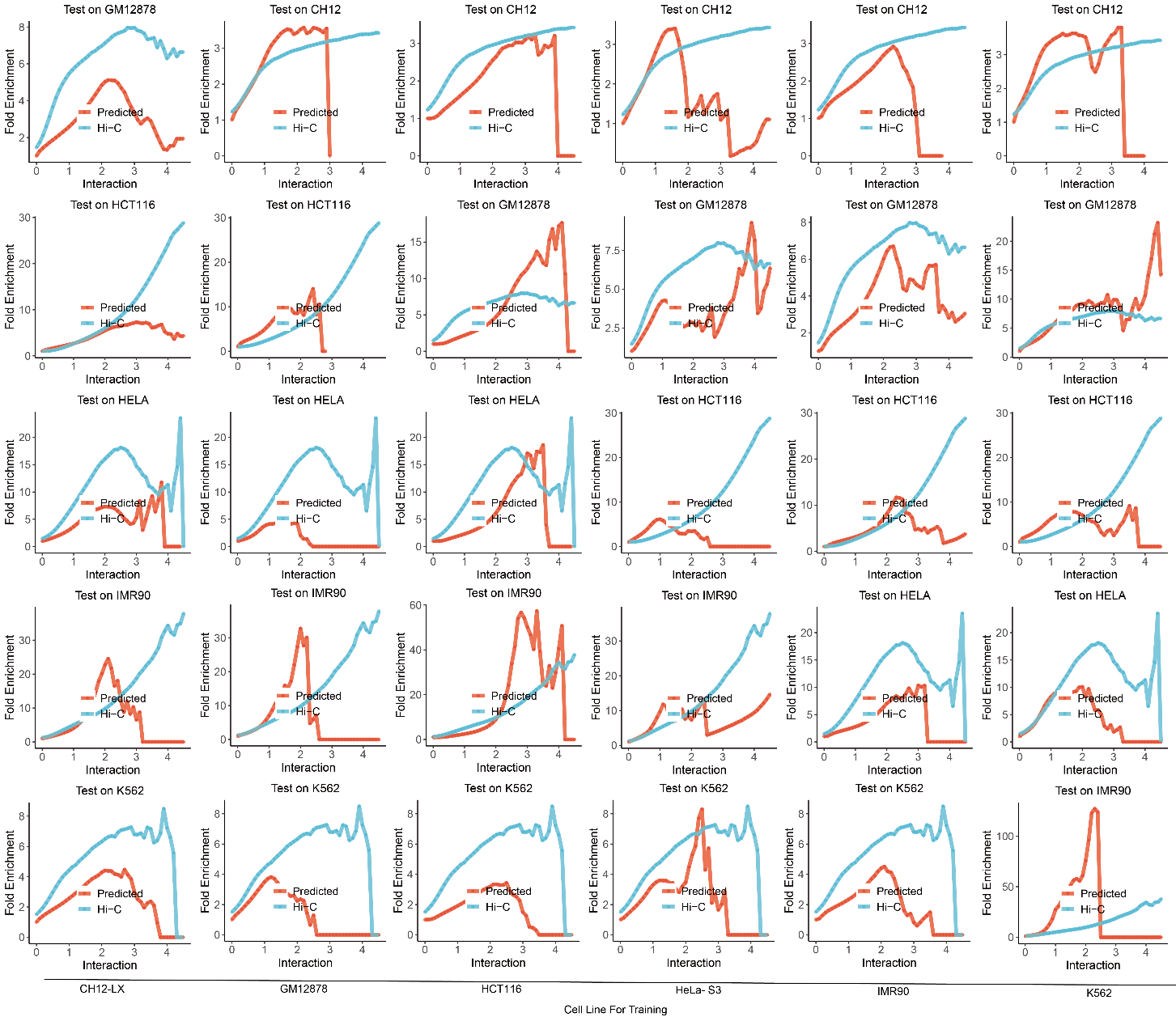


Supplementary Figure 19 The fold-of-enrichment for known enhancer-gene interactions corresponding to different thresholding of either the predicted scores based on “DynamicEdgeConv” model or the true Hi-C scores. In all cases, the predictions for each given cell line were based on model of which the parameters were trained by a different one.


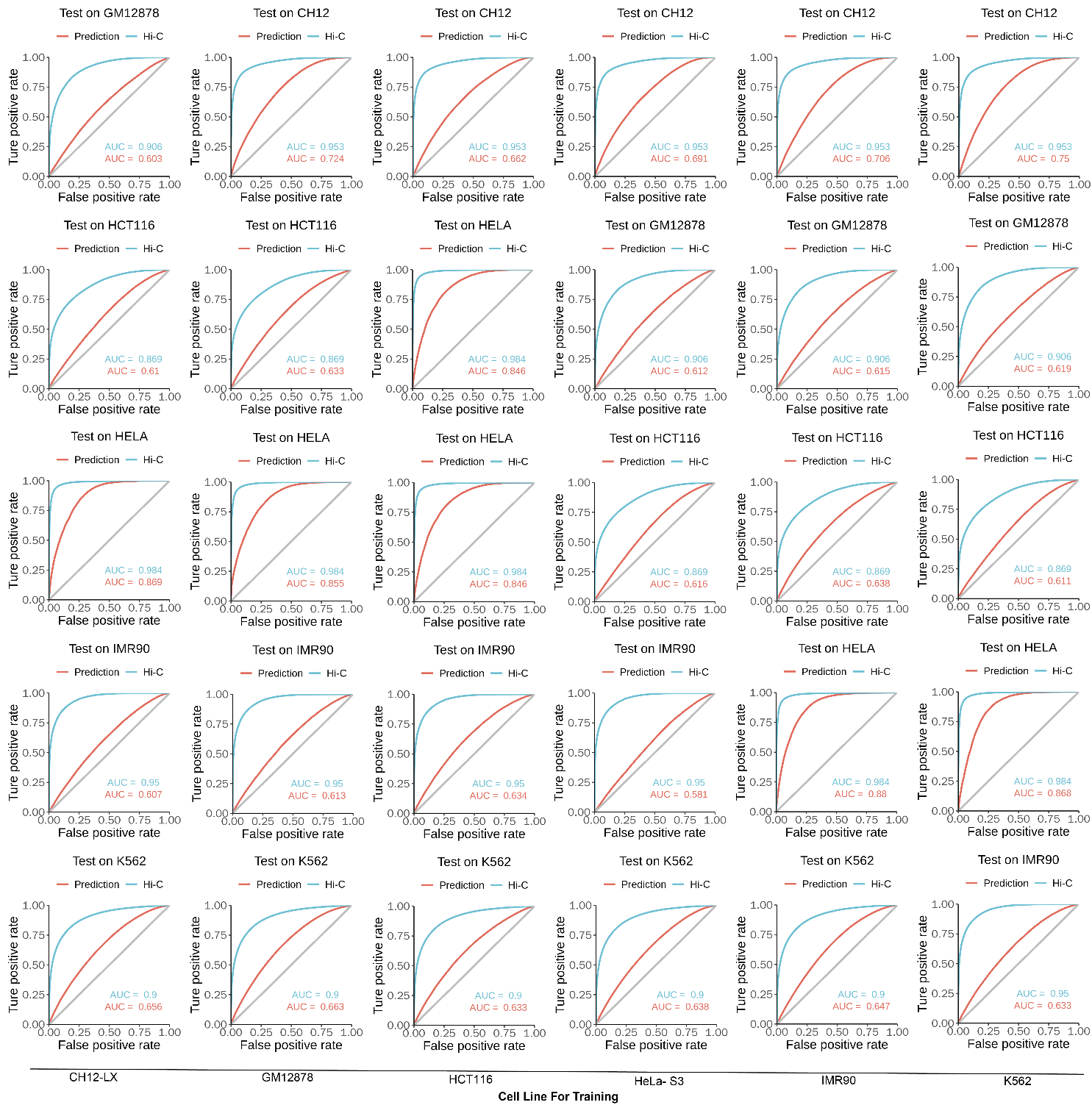


Supplementary Figure 20 The ROC curves for classification of within- and between-TAD chromatin interactions using either the predicted scores by the “DynamicEdgeConv” model or the true Hi-C scores. The model parameters are trained using data from a different cell line. The reference TADs were inferred based on Arrowhead.


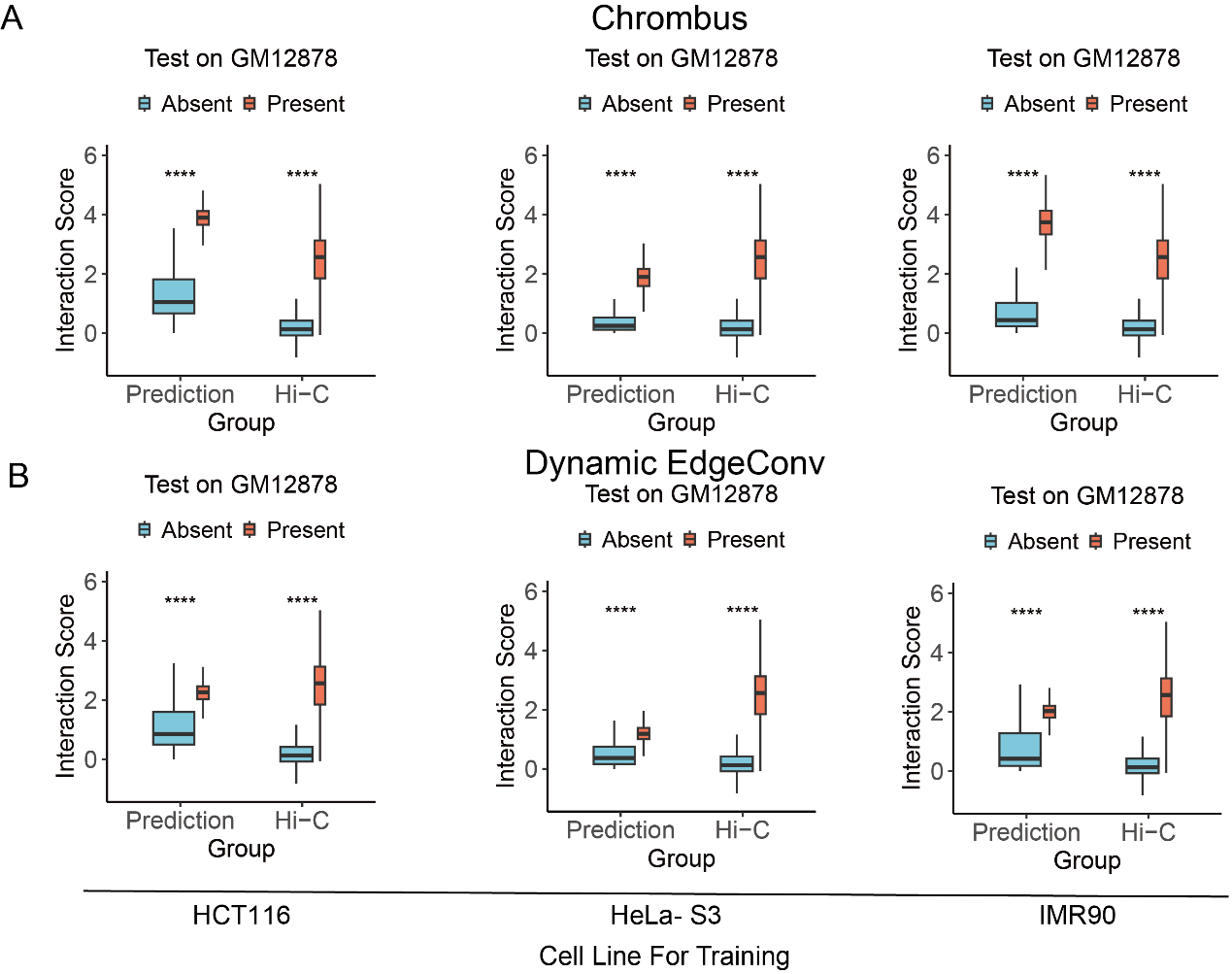


Supplementary Figure 21 Comparison of the interaction scores by Chrombus’ and DynamicEdgeConv’s prediction and Hi-C for segment pairs encompassing eQTL interaction and those do not in GM12878 cell line. In all cases, the model parameters were trained by data from a different cell line. A. The interaction scores based on Chrombus’ preddiction. B. The interaction scores based on DynamicEdgeConv model.


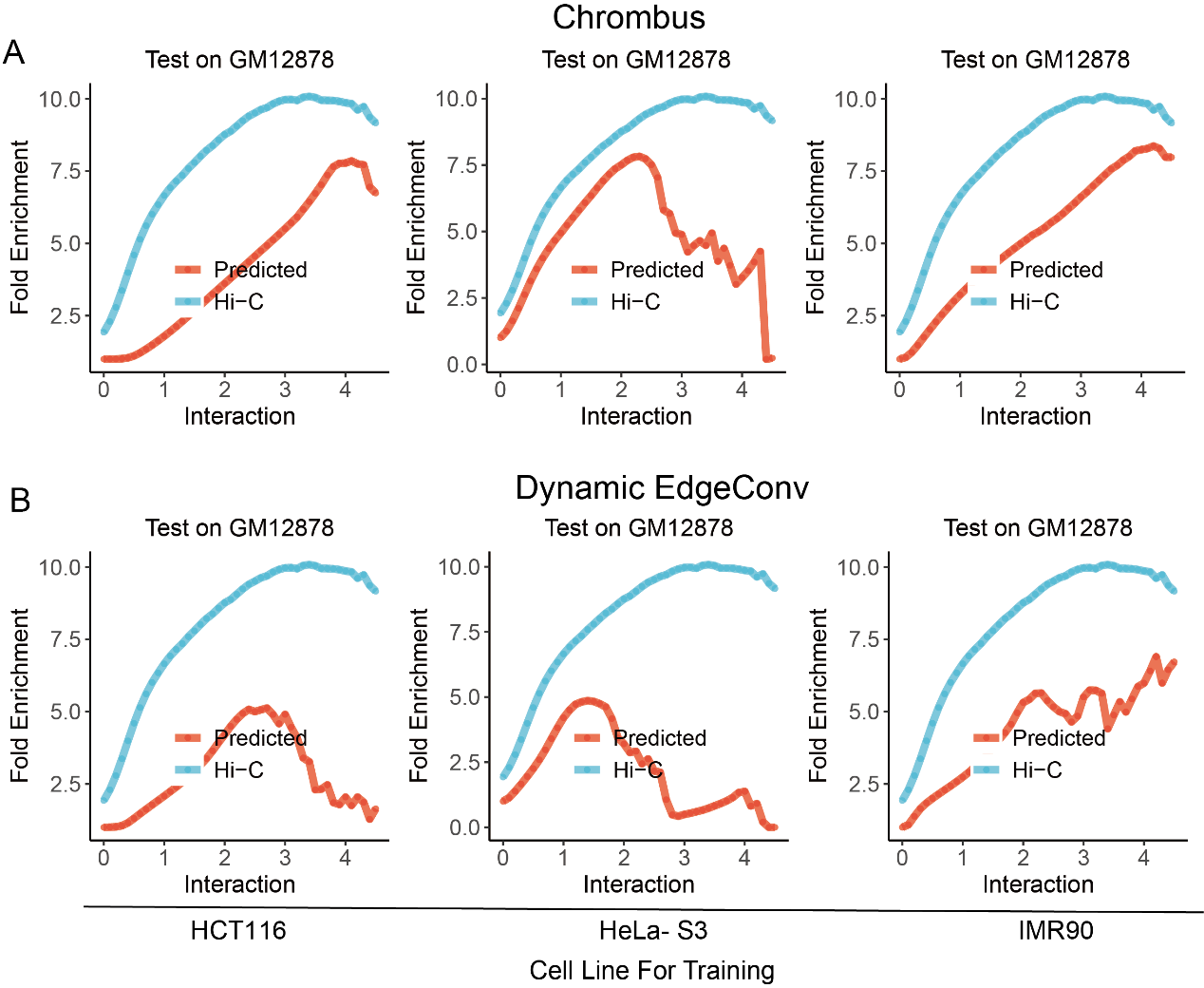


Supplementary Figure 22 Comparison of fold-of-enrichment for known eQTL-eGENE corresponding to different thresholding of the predicted scores and Hi-C scores. In all cases, the model parameters were trained by data from a different cell line. A. The predictions were obtained from Chrombus. B. The predictions were obtained from baseline model DynamicEdgeConv.


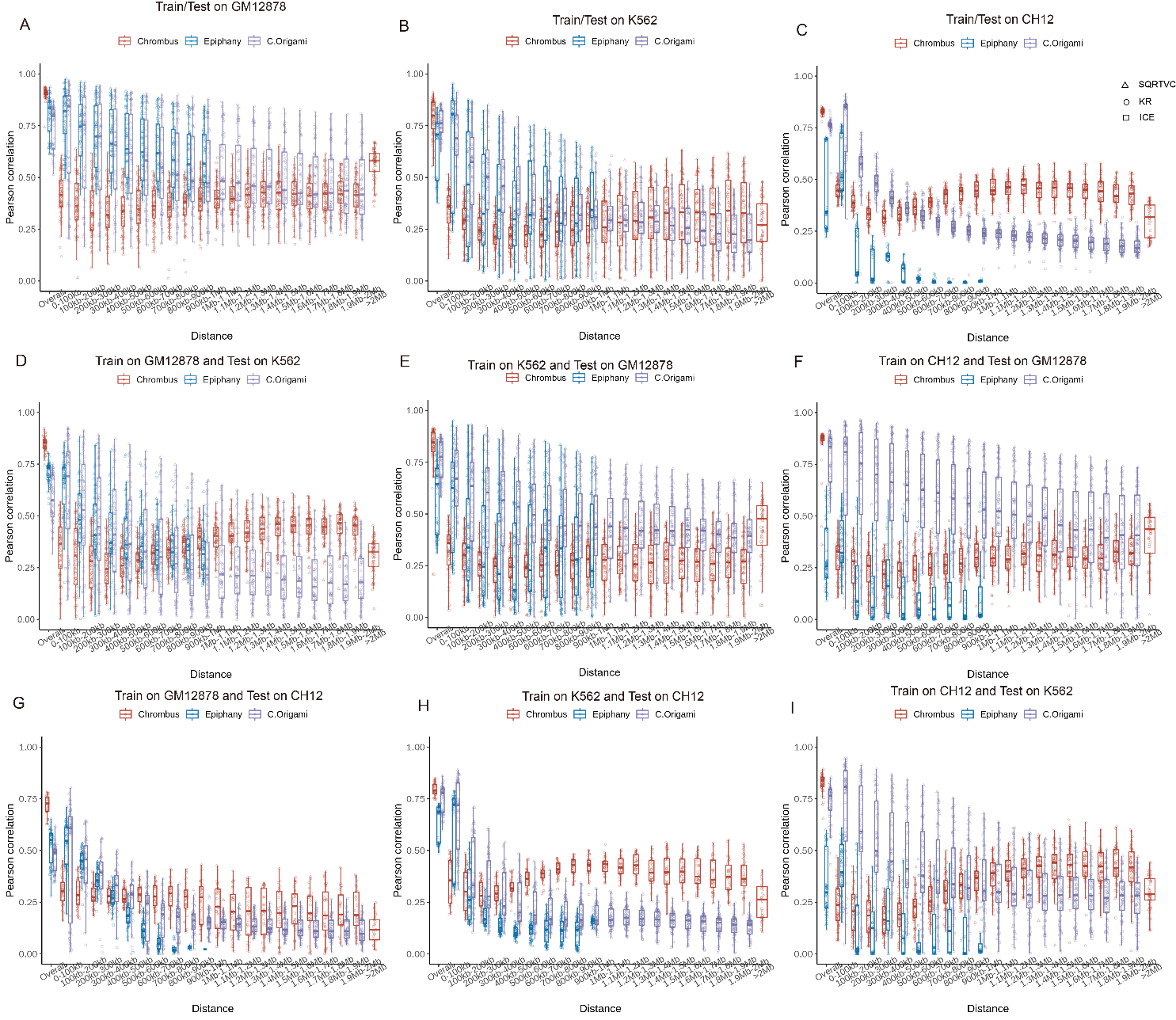


Supplementary Figure 23 Comparison of the predictive performance for chromatin interactions by Chrombus, Epiphany and C.Origami. The interactions are grouped by distance intervals of 100kb. A-C: The correlation between prediction scores of chromatin interactions and the true Hi-C score for all interactions (overall), and interactions at each intervals. The models were trained and tested on GM12878 (A), K562 (B) and CH12 (C). D-I: The correlation between prediction scores and the true Hi-C scores by the models trained on one cell line and tested on another, including: trained on GM12878 and tested on K562 (D), trained on K562 and tested on GM12878 (E), trained on CH12 and tested on GM12878 (F), trained on GM12878 and tested on CH12 (G), trained on K562 and tested on CH12 (H), trained on CH12 and tested on K562 (H). In all cases, Chrombus showed comparable predictive power for chromatin interactions over 2Mb.


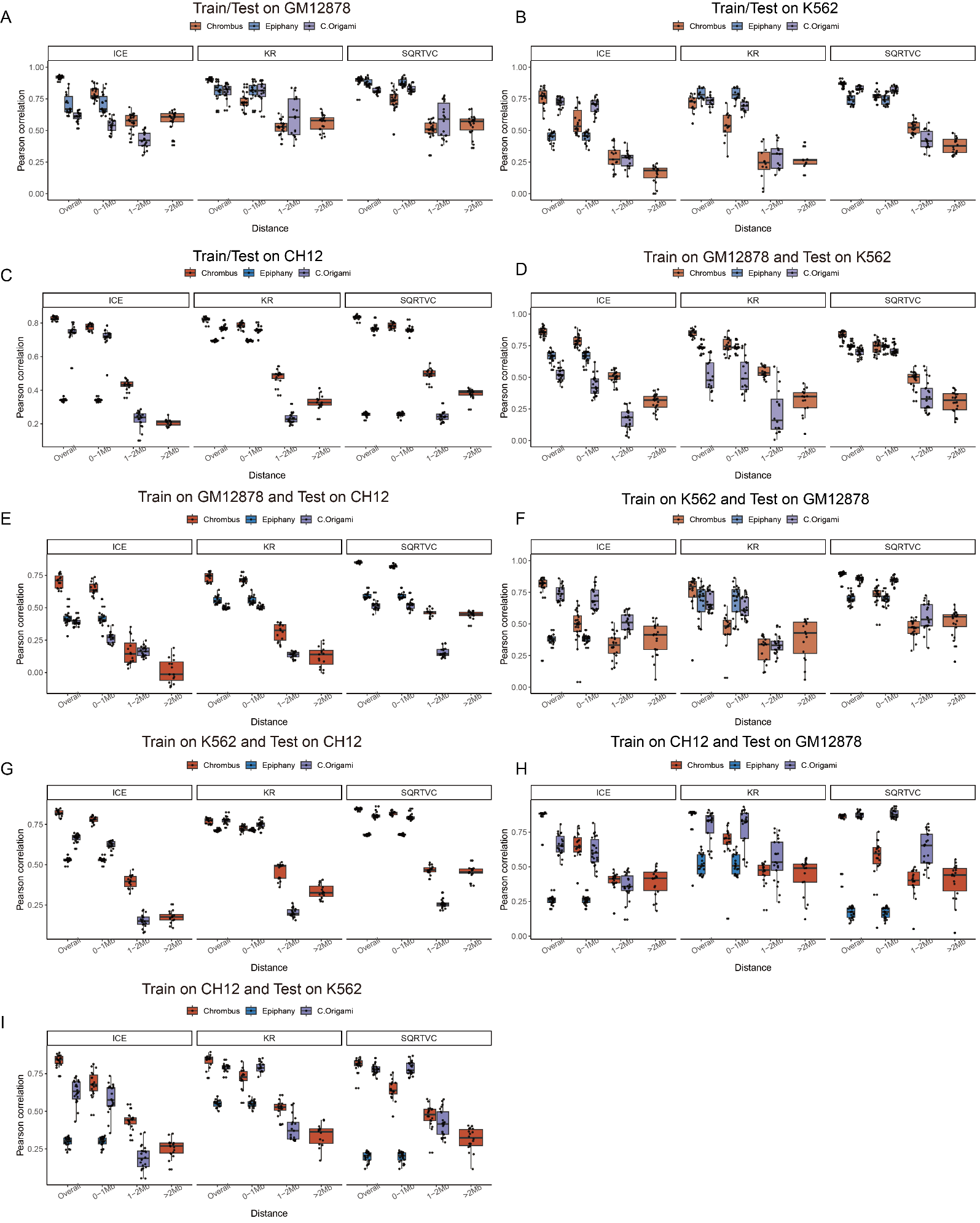


Supplementary Figure 24 Comparison of the predictive performance for chromatin interactions by Chrombus, Epiphany and C.Origami. The box is split by Hi-C normalization methods: ICE, KR and SQRTVC. A-I. The correlation between prediction scores by the models and the true Hi-C scores.


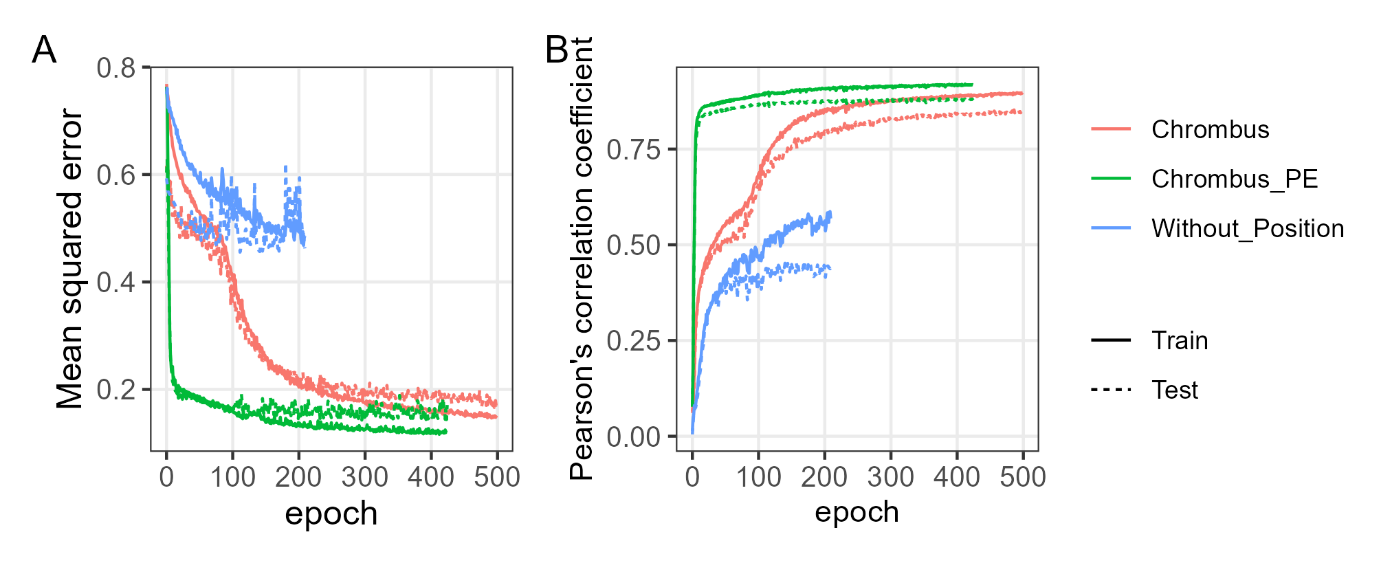


Supplementary Figure 25 Comparison of Chrombus, Chrombus with rank-based positional encoding (Chrombus_PE) and Chrombus without absolute- or rank-based position (Without_Position). A-B: Training loss and Pearson correlation coefficient of three models during maximum of 500 epochs.


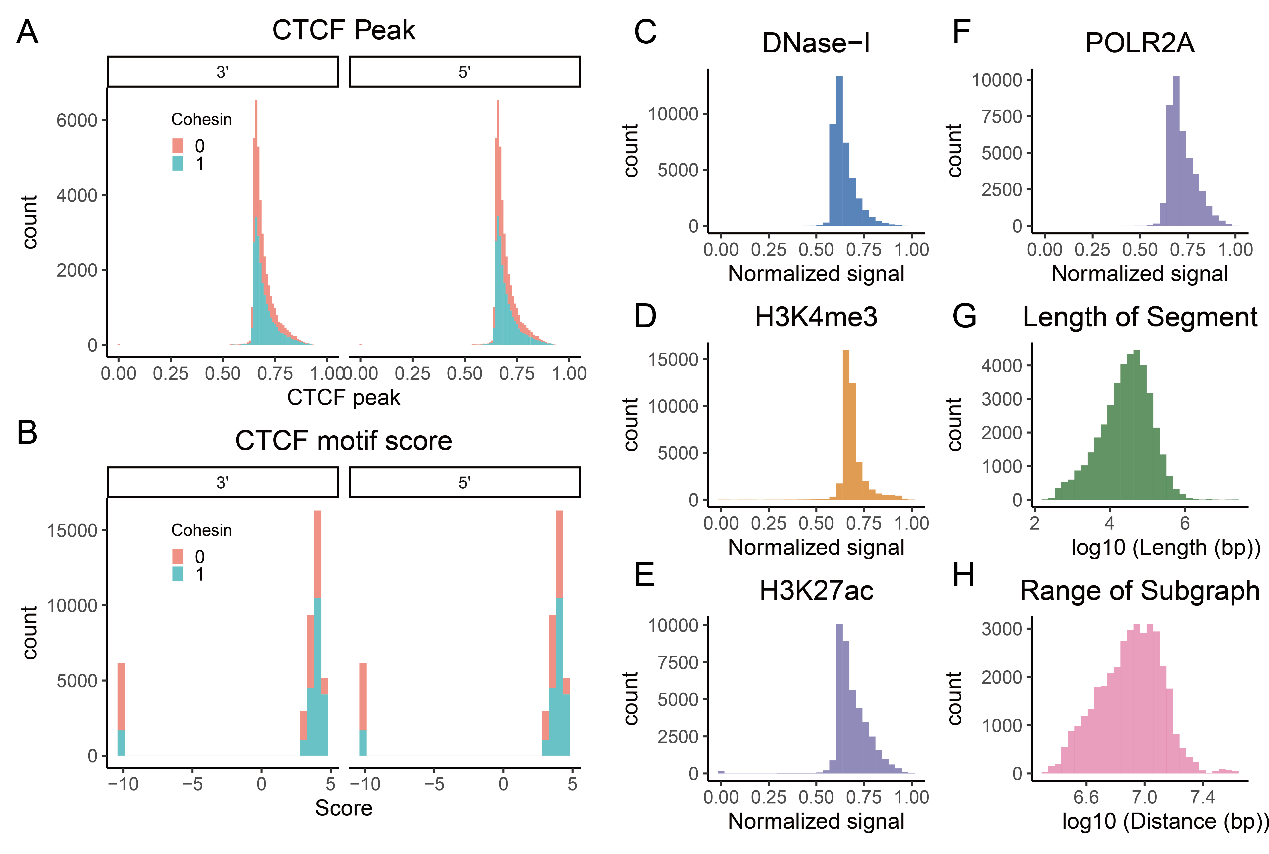


Supplementary Figure 26 Distribution of the input node features of GM12878 cell line. A: Distribution of normalized CTCF-binding strength at both ends of the segment, denotes as 5’ and 3’. Group of “1” (blue) indicated that cohesin (RAD21) binding peak was in vicinity of the segment (within a 500bp), and segments labeled as "0" indicate the absence of a nearby cohesin peak. B: Distribution of normalized CTCF motif scores at both ends of the segment, also grouped by cohesion status. C-F: Distribution of open chromatin (DNase-I) signals, H3K27ac, H3K4me3 and POLR2A within each segment, which were scaled to the range of [0, 1]. G: Distribution of length of segment in GM12878 cell line. H: Range of each subgraph of 128 segments in GM12878 cell line.


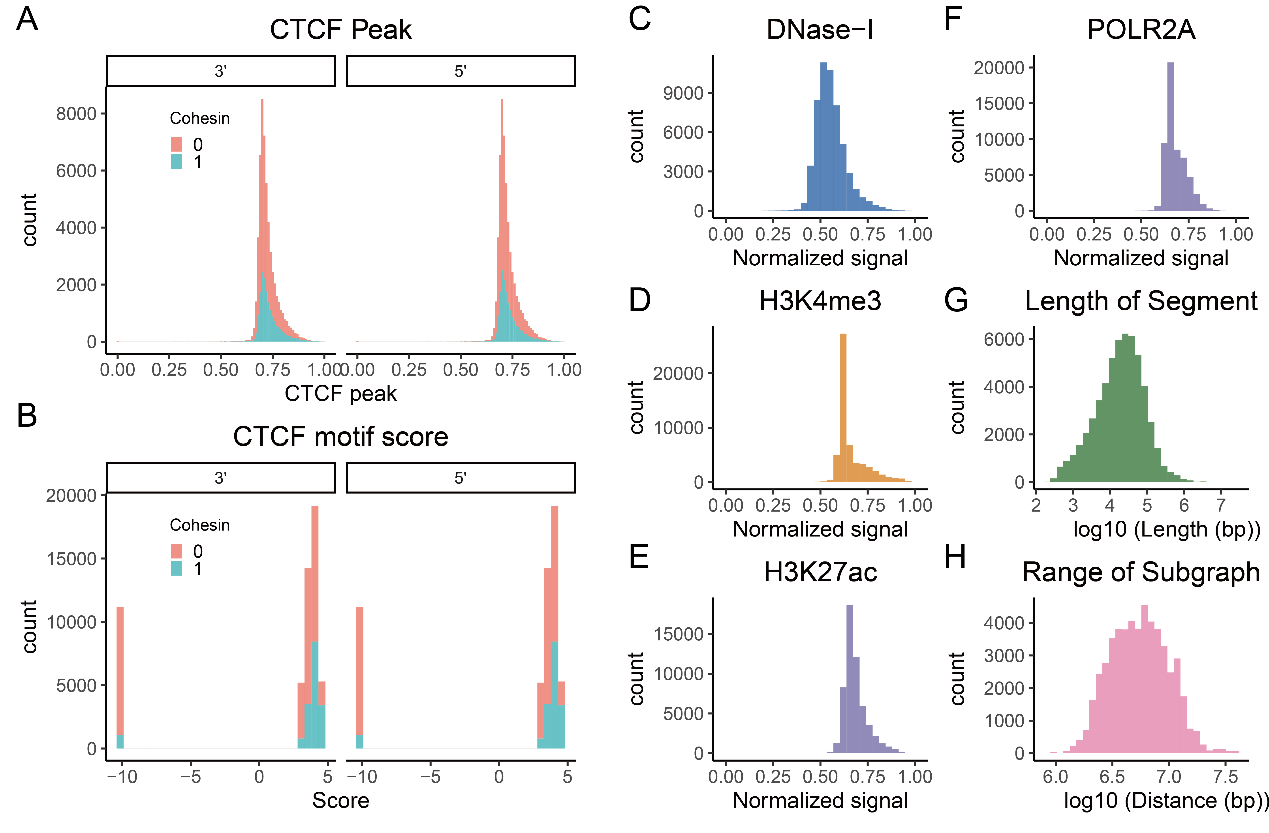


Supplementary Figure 27 Distribution of the input node features of K562 cell line. A: Distribution of normalized CTCF-binding strength at both ends of the segment, denotes as 5’ and 3’. Group of “1” (blue) indicated that cohesin (RAD21) binding peak was in vicinity of the segment (within a 500bp), and segments labeled as "0" indicate the absence of a nearby cohesin peak. B: Distribution of normalized CTCF motif scores at both ends of the segment, also grouped by cohesion status. C-F: Distribution of open chromatin (DNase-I) signals, H3K27ac, H3K4me3 and POLR2A within each segment, which were scaled to the range of [0, 1]. G: Distribution of length of segment in K562 cell line. H: Range of each subgraph of 128 segments in K562 cell line.


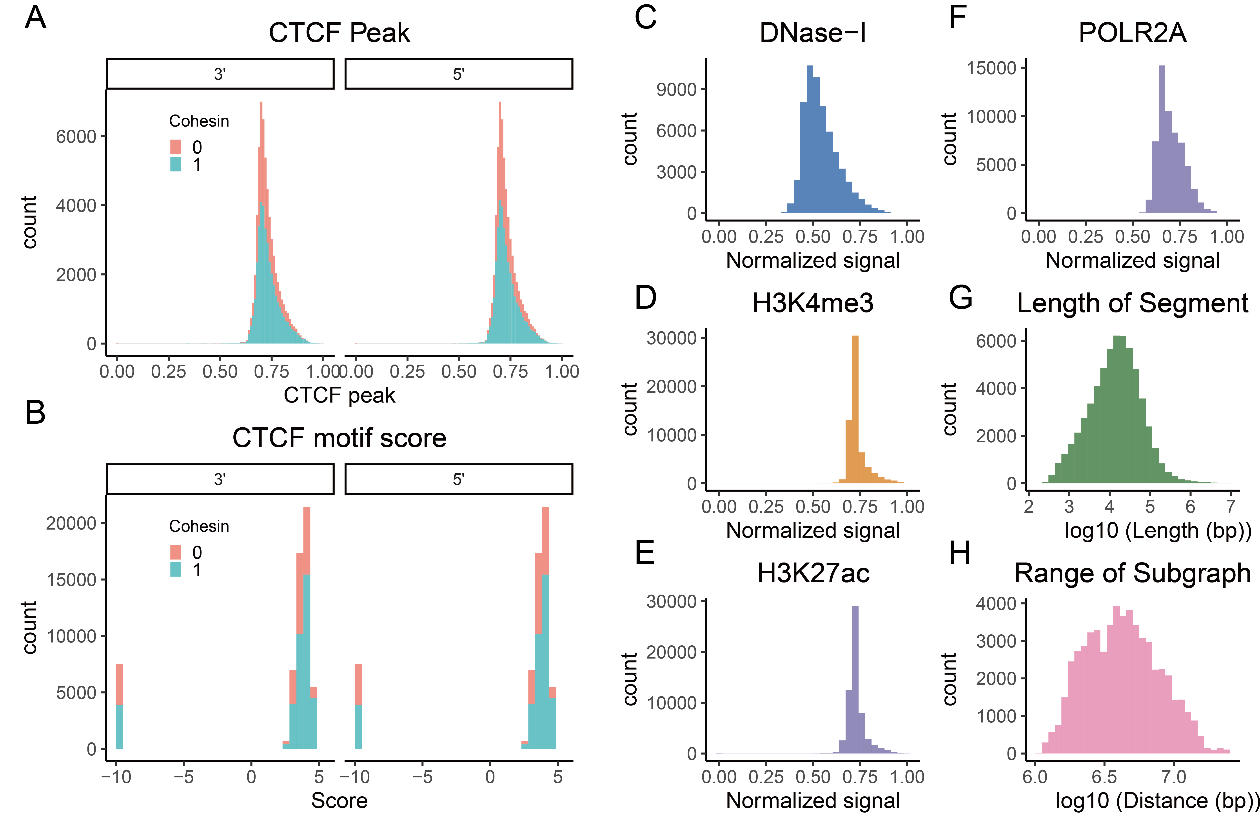


Supplementary Figure 28 Distribution of the input node features of CH12 cell line. A: Distribution of normalized CTCF-binding strength at both ends of the segment, denotes as 5’ and 3’. Group of “1” (blue) indicated that cohesin (RAD21) binding peak was in vicinity of the segment (within a 500bp), and segments labeled as "0" indicate the absence of a nearby cohesin peak. B: Distribution of normalized CTCF motif scores at both ends of the segment, also grouped by cohesion status. C-F: Distribution of open chromatin (DNase-I) signals, H3K27ac, H3K4me3 and POLR2A within each segment, which were scaled to the range of [0, 1]. G: Distribution of length of segment in CH12 cell line. H: Range of each subgraph of 128 segments in CH12 cell line.


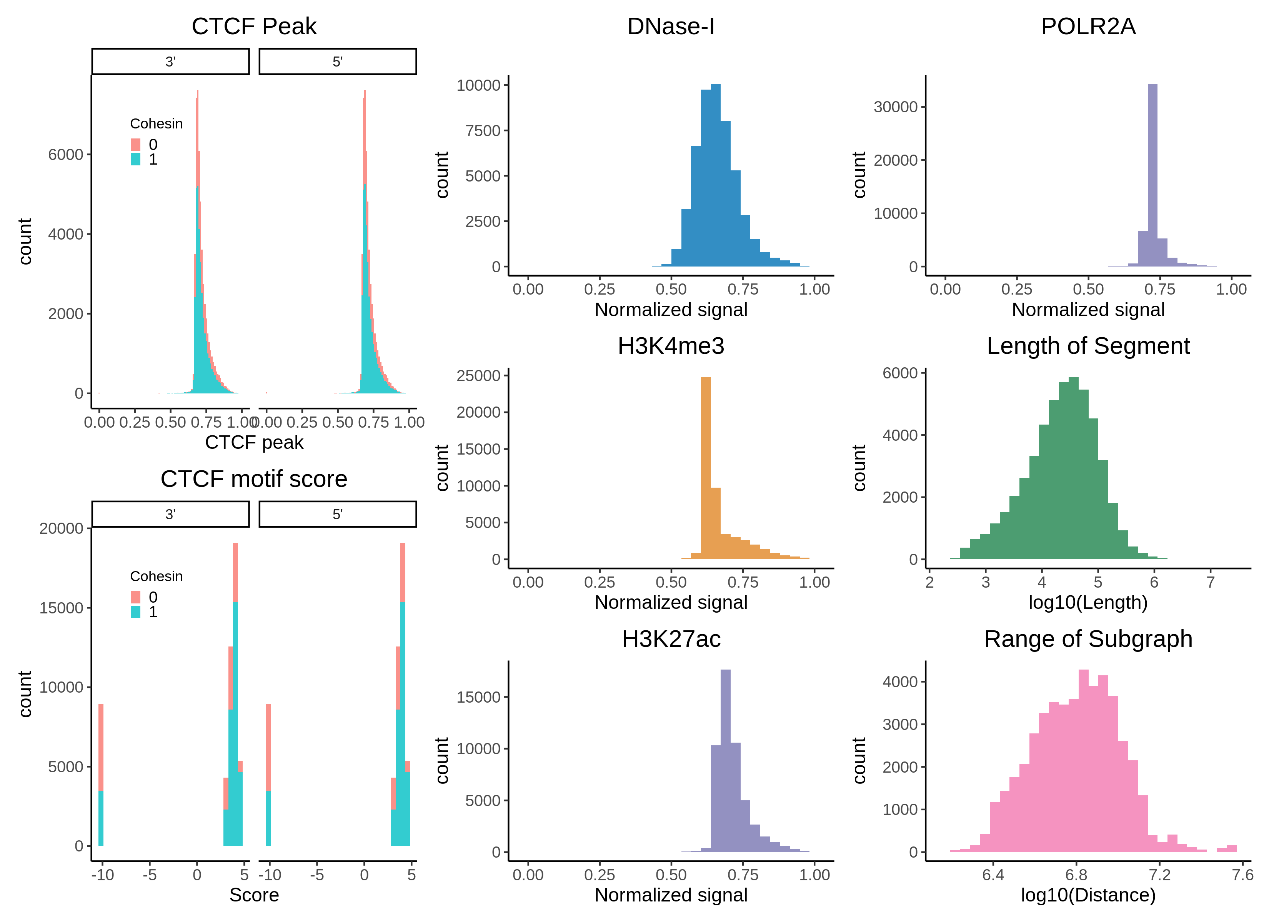


Supplementary Figure 29 Distribution of the input node features of HCT116 cell line. A: Distribution of normalized CTCF-binding strength at both ends of the segment, denotes as 5’ and 3’. Group of “1” (blue) indicated that cohesin (RAD21) binding peak was in vicinity of the segment (within a 500bp), and segments labeled as "0" indicate the absence of a nearby cohesin peak. B: Distribution of normalized CTCF motif scores at both ends of the segment, also grouped by cohesion status. C-F: Distribution of open chromatin (DNase-I) signals, H3K27ac, H3K4me3 and POLR2A within each segment, which were scaled to the range of [0, 1]. G: Distribution of length of segment in HCT116 cell line. H: Range of each subgraph of 128 segments in HCT116 cell line.


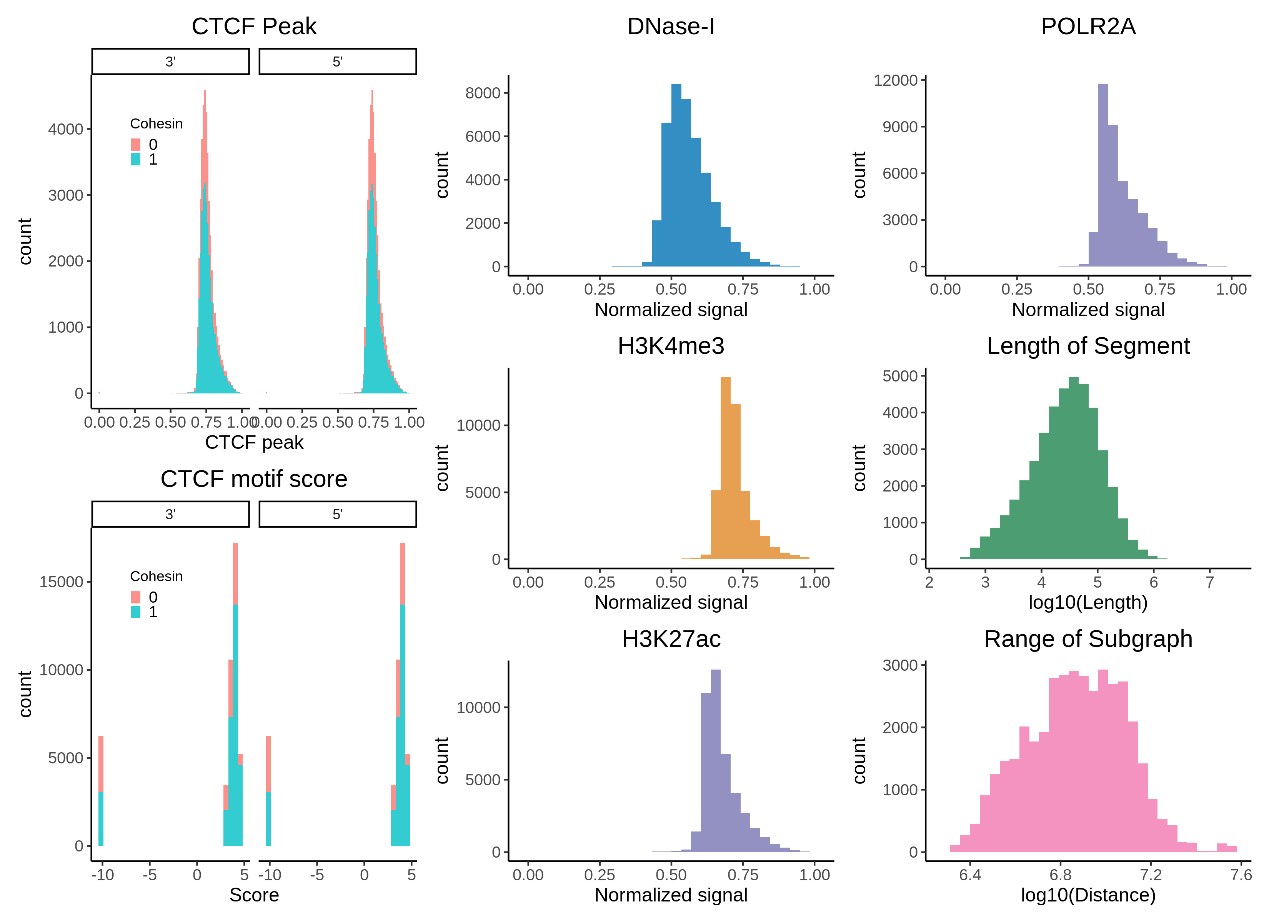


Supplementary Figure 30 Distribution of the input node features of HeLa-S3 cell line. A: Distribution of normalized CTCF-binding strength at both ends of the segment, denotes as 5’ and 3’. Group of “1” (blue) indicated that cohesin (RAD21) binding peak was in vicinity of the segment (within a 500bp), and segments labeled as "0" indicate the absence of a nearby cohesin peak. B: Distribution of normalized CTCF motif scores at both ends of the segment, also grouped by cohesion status. C-F: Distribution of open chromatin (DNase-I) signals, H3K27ac, H3K4me3 and POLR2A within each segment, which were scaled to the range of [0, 1]. G: Distribution of length of segment in HeLa-S3 cell line. H: Range of each subgraph of 128 segments in HeLa-S3 cell line.


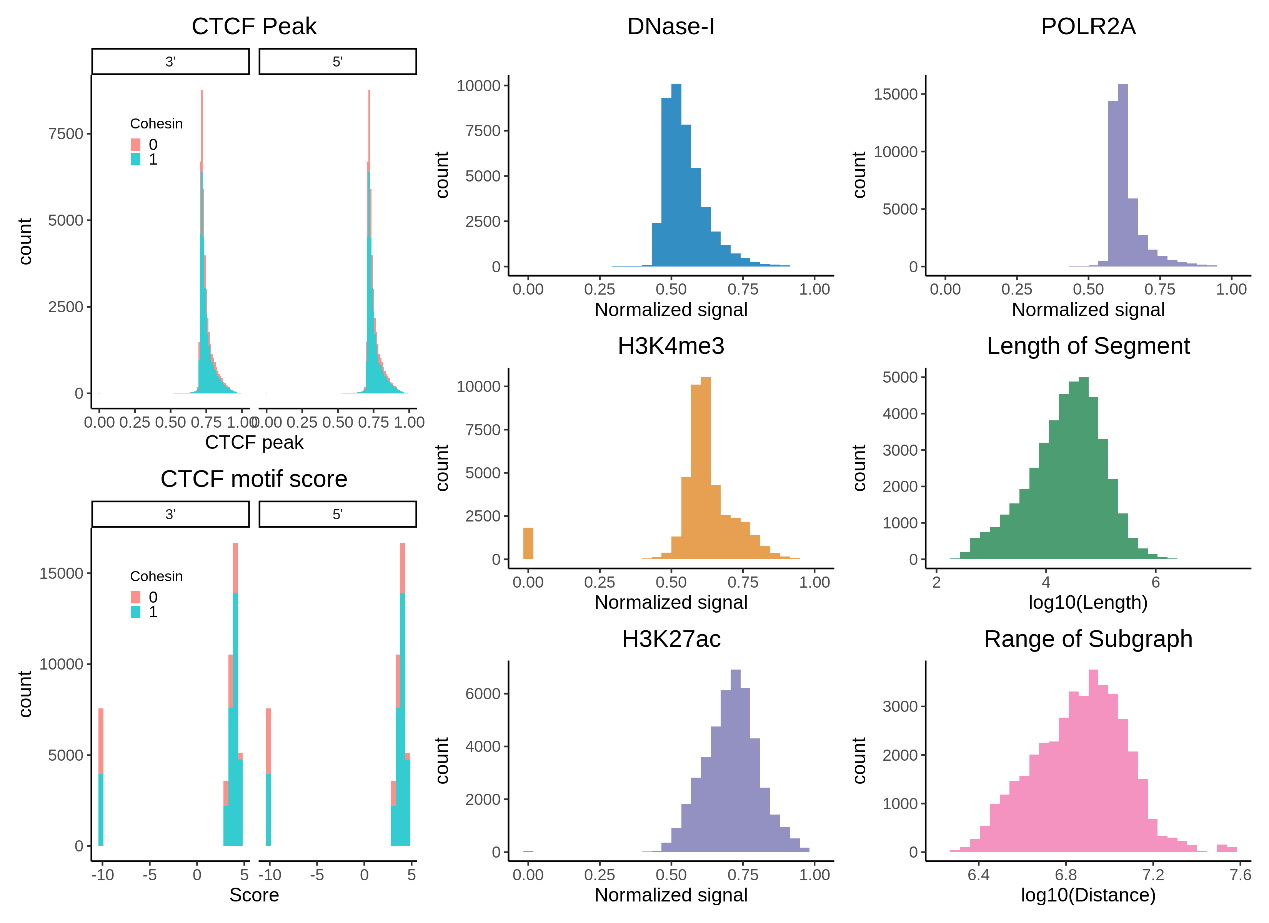


Supplementary Figure 31 Distribution of the input node features of IMR90 cell line. A: Distribution of normalized CTCF-binding strength at both ends of the segment, denotes as 5’ and 3’. Group of “1” (blue) indicated that cohesin (RAD21) binding peak was in vicinity of the segment (within a 500bp), and segments labeled as "0" indicate the absence of a nearby cohesin peak. B: Distribution of normalized CTCF motif scores at both ends of the segment, also grouped by cohesion status. C-F: Distribution of open chromatin (DNase-I) signals, H3K27ac, H3K4me3 and POLR2A within each segment, which were scaled to the range of [0, 1]. G: Distribution of length of segment in IMR90 cell line. H: Range of each subgraph of 128 segments in IMR90 cell line.


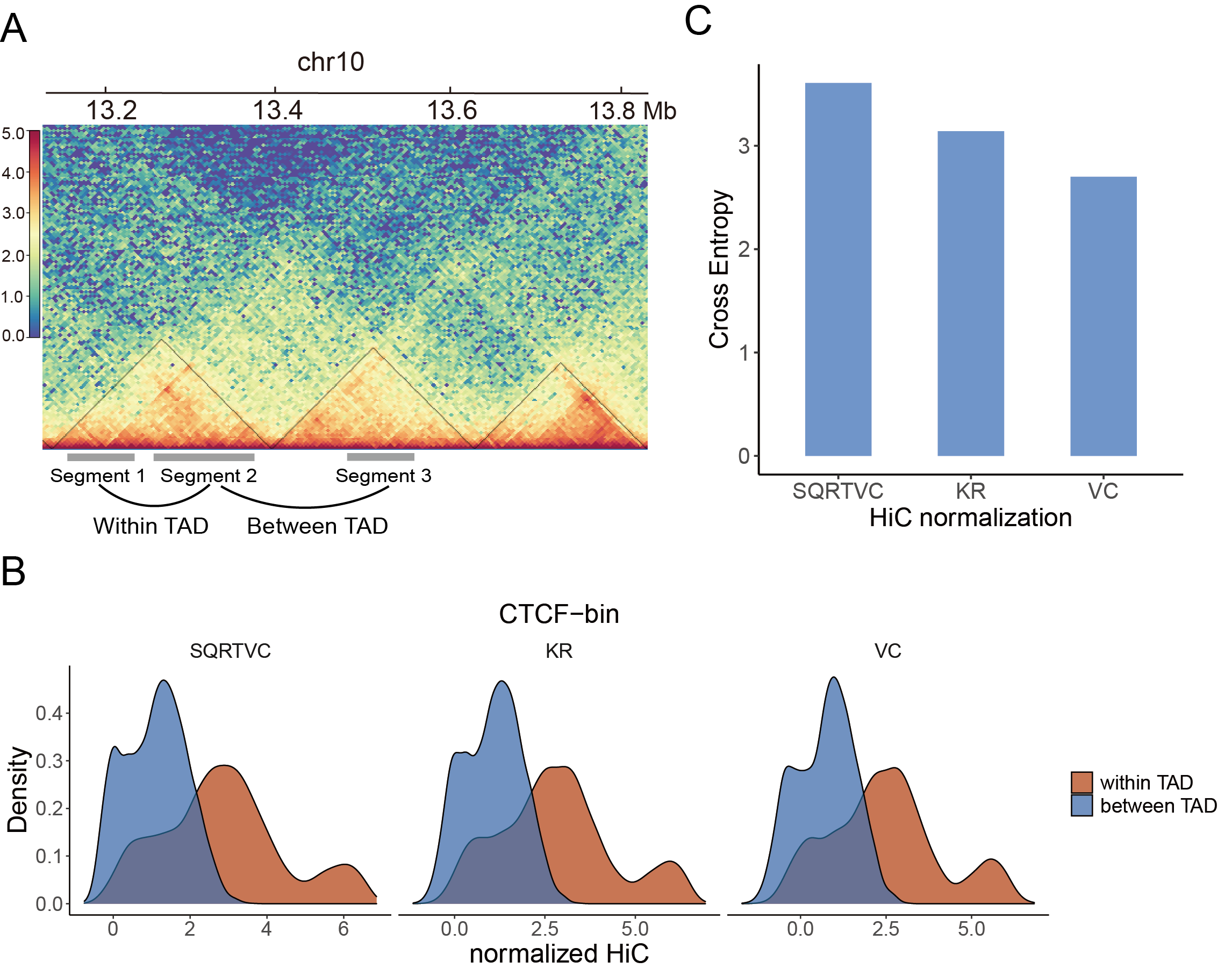


Supplementary Figure 32 Choice of the normalization methods for Hi-C data influence the predictive performance of Chrombus. A: illustration of interactions within and between TADs. B: Distribution of processed Hi-C scores for within- and between-TAD interactions based on three normalization methods: SQRTVC, KR and VC. C: The discriminative power of processed Hi-C scores for within- and between-TAD interactions based on three normalization methods were evaluated using Cross-Entropy, showing the normalization methods best representing TAD structure.


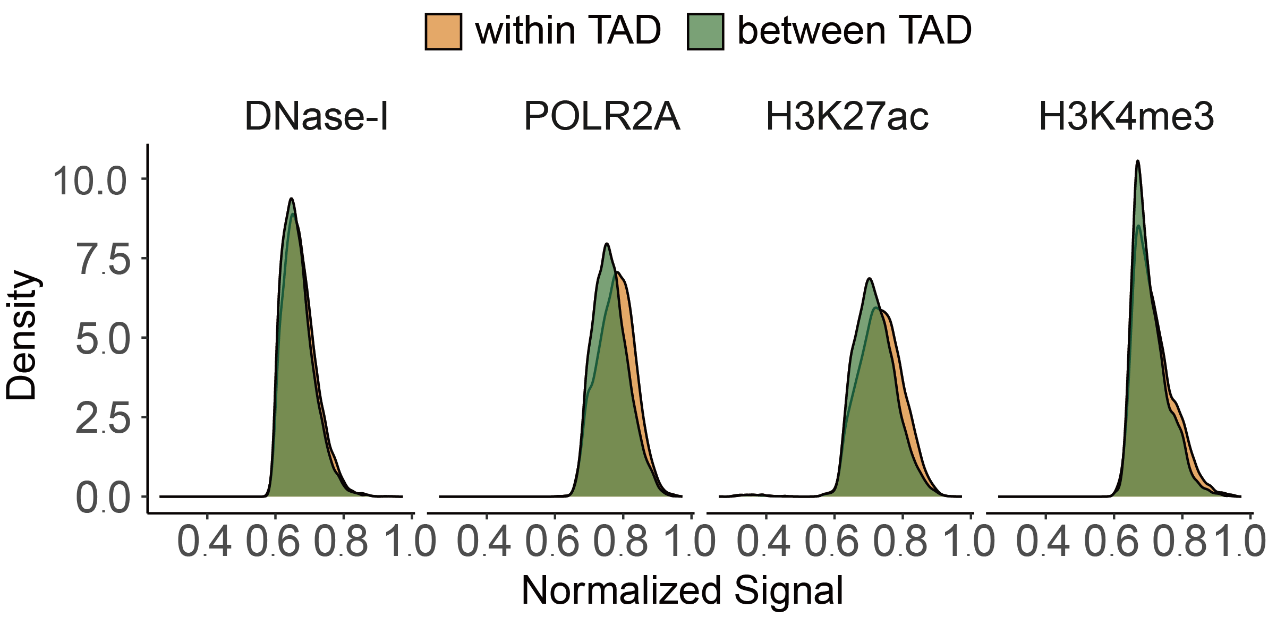


Supplementary Figure 33 Distribution of signal strength of four input features: DNase-I, POLR2A, H3K27ac, H3K4me3, for within- and between-TAD chromatin interactions.

Reference:

1. Ramirez, F., et al., High-Affinity Sites Form an Interaction Network to Facilitate Spreading of the MSL Complex across the X Chromosome in Drosophila. Mol Cell, 2015. 60(1): p. 146-62.

2. Consortium, G.T., The Genotype-Tissue Expression (GTEx) project. Nat Genet, 2013. 45(6): p. 580-5.

3. Karatzoglou, A., et al., kernlab - An S4 Package for Kernel Methods in R. Journal of Statistical Software, 2004. 11(9): p. 1 - 20.

4. Csardi G, N.T., The igraph software package for complex network research. InterJournal, Complex Systems, 2006: p. 1695.
